# *Plasmodium falciparum* Gametocyte Development 1 (*Pfgdv1*) and Gametocytogenesis Early Gene Identification and Commitment to Sexual Development

**DOI:** 10.1371/journal.ppat.1002964

**Published:** 2012-10-18

**Authors:** Saliha Eksi, Belinda J. Morahan, Yoseph Haile, Tetsuya Furuya, Hongying Jiang, Omar Ali, Huichun Xu, Kirakorn Kiattibutr, Amreena Suri, Beata Czesny, Adebowale Adeyemo, Timothy G. Myers, Jetsumon Sattabongkot, Xin-zhuan Su, Kim C. Williamson

**Affiliations:** 1 Department of Biology, Loyola University Chicago, Chicago, Illinois, United States of America; 2 Laboratory of Malaria and Vector Research, National Institute of Allergy and Infectious Diseases, National Institutes of Health, Bethesda, Maryland, United States of America; 3 Center for Research on Genomics and Global Health, Inherited Disease Research Branch, National Human Genomics Research Institute, National Institutes of Health, Bethesda, Maryland, United States of America; 4 Department of Entomology, U.S. Army Medical Component, Armed Forces Research Institute of Medical Sciences, Bangkok, Thailand; 5 Genomic Technologies Section, Research Technologies Branch, National Institute of Allergy and Infectious Diseases, National Institutes of Health, Bethesda, Maryland, United States of America; Weill Cornell Medical College, United States of America

## Abstract

Malaria transmission requires the production of male and female gametocytes in the human host followed by fertilization and sporogonic development in the mosquito midgut. Although essential for the spread of malaria through the population, little is known about the initiation of gametocytogenesis *in vitro* or *in vivo*. Using a gametocyte-defective parasite line and genetic complementation, we show that *Plasmodium falciparum* gametocyte development 1 gene (*Pfgdv1)*, encoding a peri-nuclear protein, is critical for early sexual differentiation. Transcriptional analysis of *Pfgdv1* negative and positive parasite lines identified a set of gametocytogenesis early genes (*Pfge*) that were significantly down-regulated (>10 fold) in the absence of *Pfgdv1* and expression was restored after *Pfgdv1* complementation. Progressive accumulation of *Pfge* transcripts during successive rounds of asexual replication in synchronized cultures suggests that gametocytes are induced continuously during asexual growth. Comparison of *Pfge* gene transcriptional profiles in patient samples divided the genes into two groups differing in their expression in mature circulating gametocytes and providing candidates to evaluate gametocyte induction and maturation separately *in vivo*. The expression profile of one of the early gametocyte specific genes, *Pfge1*, correlated significantly with asexual parasitemia, which is consistent with the ongoing induction of gametocytogenesis during asexual growth observed *in vitro* and reinforces the need for sustained transmission-blocking strategies to eliminate malaria.

## Author Summary

As malaria control efforts move toward eradication it becomes increasingly important to develop interventions that block transmission. Consequently, advances are needed in our understanding of the production of gametocytes, which are required to transmit the disease. This report provides a first view of the initial stages of gametocytogenesis *in vitro* and *in vivo* and demonstrates that during each asexual replication cycle a subpopulation of parasites convert to gametocyte development providing a long transmission window. We also identify a gene that is critical for gametocyte production, *P. falciparum* gametocyte development 1 (*Pfgdv1*) and a set of genes specifically expressed during early gametocytogenesis in *P. falciparum* (*Pfge* genes). The expression profile and peri-nuclear location of *Pfgdv1* in a subpopulation of schizonts is consistent with a role in an early step in gametocytogenesis. The RNA levels of *Pfgdv1* and the *Pfge* genes accumulated gradually over several asexual cycles *in vitro* suggesting ongoing gametocyte formation during asexual growth. The further evaluation of these genes in a cohort of malaria infected patients indicated they are good candidates for markers to distinguish ring stage parasites committed to gametocyte production from circulating mature gametocytes, allowing direct analysis of the initiation of sexual differentiation *in vivo*.

## Introduction

The spread of malaria, a disease that continues to be responsible for 200–300 million clinical cases and the deaths of ∼0.7 million people each year, requires the production of gametocytes for transmission from person to person via a mosquito [1], [2]. Importantly, it has been shown that gametocytes are not effectively cleared by common antimalarial drugs and therefore allow transmission to mosquitoes despite clearance of asexual parasites [3]. Thus, as efforts toward malaria elimination and eradication progress, the need to understand and monitor the dynamics of the infectious reservoir intensifies.

Within the human host, intraerythrocytic parasites can either replicate asexually or differentiate into a single male or female gametocyte. After release from an infected liver cell, merozoites invade red blood cells (RBC) and the resulting ring stage parasites develop into schizonts containing 16–32 merozoites that are released into the circulation and can each invade a new RBC to begin the cycle again. For transmission to mosquitoes, a subpopulation of schizonts produce merozoites that invade RBC and each differentiate over the course of 10–12 days into a single male or female gametocyte [4]–[8]. After being taken up in a blood meal by a mosquito, gametocytes are stimulated to produce gametes, mate and develop into sporozoites that are infectious to humans during subsequent blood-feeding. Despite the importance of gametocytogenesis in the propagation of infection, the nature and timing of its induction is unknown [9]–[11]. Specifically, the lack of markers for early sexual stages, gametocyte-committed schizonts and the ring-stage equivalent of sexually committed parasites has made it difficult to determine whether gametocyte production is a one-time stimulus-induced event or a continuous process with a subpopulation of parasites converting to sexual development during each cycle [10], [12], [13]. This has important ramifications for control strategies as continuous gametocyte production would significantly prolong the transmission window. In addition to the lack of early gametocytogenesis markers, the analysis of *P. falciparum* gametocyte development is complicated by sequestration of both mature asexual parasites (trophozoites and schizonts) and immature gametocytes (stages II to IV) in vasculature during human infection, which means that only rings, including those committed to sexual development, and mature gametocytes are detected in peripheral blood [13].

To better define gametocyte commitment and evaluate the timing of induction, we compared clonal parasite lines that differed in their ability to produce gametocytes to identify the genes that contribute to gametocytogenesis. Genetic complementation confirmed the important role of one of the identified genes, *P. falciparum* gametocyte development 1 gene (*Pfgdv1*) in gametocyte production and the expression of a set of *P. falciparum* gametocytogenesis early genes (*Pfge*). The transcription profiles of these genes were then used to investigate gametocyte induction through sequential asexual cycles *in vitro* demonstrating the continuous production of gametocytes. The analysis was also extended to clinical samples and suggests that there are distinct molecular signatures for mature gametocytes and early committed gametocytes in patient blood samples that could be used in future studies to differentiate gametocyte induction from maturation. This work describes the first direct analysis of this critical early step toward malaria transmission and identifies markers to continue to probe sexual differentiation *in vitro* and *in vivo*.

## Results

### Identification of an early *P. falciparum* gametocyte-specific transcriptome

**Figure 1:**
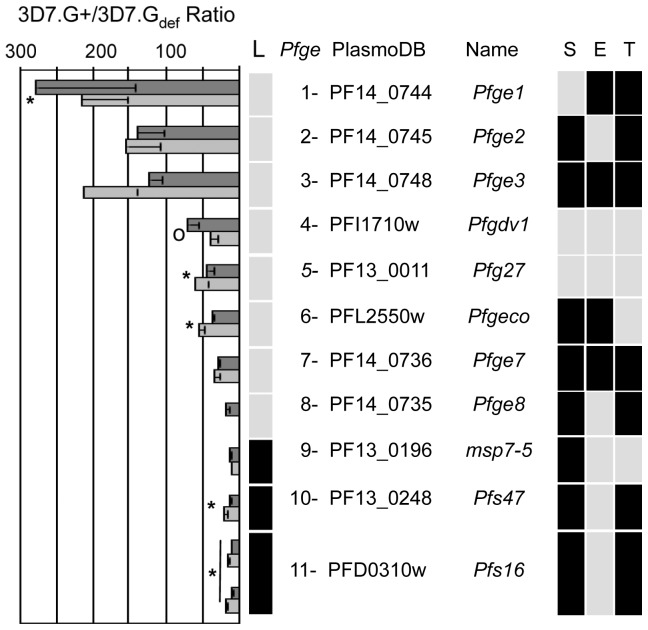
Expression analysis of the 3D7.G+ and 3D7.G_def_ clones identifies *P. falciparum* gametocytogenesis early genes (*Pfge*). Mean ratios of microarray signals (mean±SEM) obtained from the 3D7.G+ and 3D7.G_def_ clones at 1.4/0.9% parasitemia (dark gray bar) and at 5.2/5.5% parasitemia (light gray bar), respectively, for the 11 *Pfge* genes with a ratio of 3D7.G+/3D7.G_def_>10 are plotted in descending order. *Pfgdv1* (PFI1710w) had the fourth highest ratio and is indicated by an ^o^. Previously described *Pfge* genes [17], [20]–[22], [82], are denoted with an asterisk (*) and a vertical line indicates a gene represented by two oligonucleotides. *Pfge* number, PlasmoDB ID, and common name are listed on the right. The central panel is a summary of the characteristics of the *Pfgdv1*-dependent genes. The first row (L) indicates whether the gene is subtelomeric (<150 kb from the telomere) (gray square) or within a region of the chromosome that has synteny with other species (black square) [83]. The panel on the left indicates whether the gene encodes a secretory signal sequence (S, black square), PEXEL/HTS export domain (E, black square) or transmembrane domain (T, black square).

To identify genes that play an important role in the initial induction of gametocytogenesis, we compared the transcriptional profile of a set of clonal gametocyte-over producing (3D7.G+, average peak gametocytemia ± SEM, 6.97±2.72%) and gametocyte-deficient (3D7.G_def_, average peak gametocytemia ± SEM, 0.009±0.009%) parasite lines. Both lines, 3D7.G+ and 3D7.G_def_, were cloned from *P. falciparum* strain 3D7 following targeted disruption of *Pfs230* (PFB0405w) [14], [15]. RNA was isolated from tightly synchronized cultures on day 4 of gametocyte induction (at parasitemias of 1.4% for 3D7.G+ and 0.9% for 3D7.G_def_) and then again 2 days later at parasitemias of 5.2% and 5.5%, respectively, and was hybridized to a 70-mer oligonucleotide array representing 5,092 *P. falciparum* genes [16]. Both time points were early in gametocytogenesis, prior to detection of stage II gametocytes in Giemsa-stained culture smears, which in previous studies was the first gametocyte-specific time point for the identification of gametocyte-specific transcripts [17]. Using a 10 fold signal difference as a threshold, 11 genes were differentially regulated at both time points (Fig. 1) and an additional 9 genes had a≥5 fold signal difference (p<0.05) (Table S1). They include seven known gametocyte specific genes *Pfg27*, *Pfs16*, *Pfg14.744*, *Pfg14.748*, *Pfs47*, *Pfmdv1*, and *Pfgeco* [18]–[23]. These seven genes, as well as PF11_0038, PF14_0290, PF14_0588, PF14_0708 and PF14_0735 have also been identified by proteomic analysis of stage I/II gametocytes; while the remaining genes have not been associated with gametocytogenesis [24]. The differential expression patterns of the 11 genes were confirmed using reverse transcriptase-quantitative polymerase chain reaction (RT-qPCR) or northern blots and were designated as *P. falciparum* gametocytogenesis early (*Pfge*) 1 to 11 according to their 3D7.G+/3D7.G_def_ signal ratio at the first time point (Fig. 1 and Table S1). This name will be used for those genes without a common name; for example, PF14_0744 had the highest signal ratio and was named *Pfge1*. *Pfge* genes 1–8 are located in subtelomeric regions (<150 kB from the telomere) and, except for *Pfge4*, only have close orthologs (Blast E score≤10^−35^) in *P. reichenowi*. Nine of the *Pfge* genes were predicted to have signals for secretion and/or export to the erythrocyte, which was consistent with gametocyte-specific modifications of the external environment being an early step in sexual differentiation.

### Defective gametocytogenesis is linked to deletion of a locus encoding *Pfgdv1*

**Figure 2:**
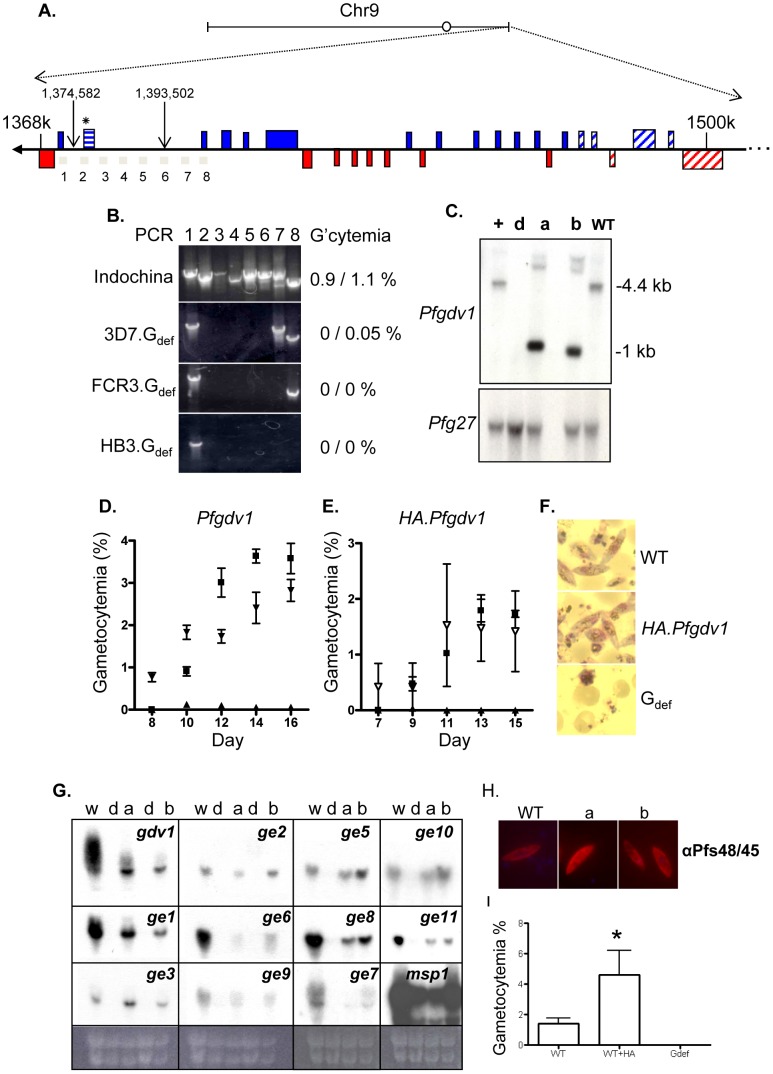
Identification of *Plasmodium falciparum* gametocyte development 1 gene (*Pfgdv1*). A) Schematic of chromosome 9 (1–1,541,723 bp) showing the segment deleted (arrows) in the 3D7.G_def_ clone. For orientation, the putative centromere is indicated by an O and the 1,500,000 bp position is marked (1500 k). Colored boxes indicate the location of annotated genes: blue, genes transcribed toward the telomere; red, transcribed toward the centromere; horizontal stripes, *Pfgdv1;* diagonal stripes, *var* and *rifins*. The 70-mer oligonucleotide that identified the chromosome 9 deletion in 3D7.G_def_ parasites is indicated by an asterisk (*). The numbered gray bars below the line indicate the positions of the eight PCR products used to map the chromosome 9 deletion. B) Amplification products from chromosome 9 using Indochina, 3D7.G_def_, FCR3.G_def_, or HB3.G_def_ gDNA as a template. Peak gametocytemias attained in two independent experiments are indicated on the right of the ethidium bromide-stained agarose gel of the PCR products generated using the eight primer pairs (1–8). C) Southern blot of *Bsa*BI-digested gDNA from 3D7.G+ (+), 3D7.G_def_ (d), 3D7.G_def_ +*Pfgdv1* (a), 3D7.G_def_ +HA.*Pfgdv1* (b) and the parental 3D7 parasites (WT). Digested gDNA was probed with *Pfgdv1* (bp 1423–1800) or *Pfg27* (bp 1–654). D–E) Gametocyte production in WT (black square), 3D7.G_def_ (black triangle), complemented line a, 3D7.G_def_ +*Pfgdv1* (Panel D, black inverted triangle) and line b, 3D7.G_def_ +HA.*Pfgdv1* (Panel E, unfilled inverted triangle). Cultures set up at 0.1% asexual parasitemia on day 0 were followed for gametocyte production by Giemsa-stained smears for the next 16 days. Mean gametocytemia and standard deviation of two (D) or three (E) independent experiments are shown (*P*<0.003 by linear regression analysis). F) Giemsa-stained smear of parasitized erythrocytes purified on a 16% Nycodenz cushion from day 16 gametocyte cultures of WT, 3D7.G_def_ +HA.*Pfgdv1* (HA.*Pfgdv1*), and 3D7.G_def_ (G_def_) lines. G) Northern blots of RNA harvested from WT 3D7 (w), 3D7.G_def_ (d), 3D7.G_def_ *+Pfgdv1* (a), and 3D7.G_def_ +HA.*Pfgdv1* (b) complemented lines were hybridized with probes corresponding to *Pfgdv1* (*gdv1*), *Pfge* genes (*ge1–3, 5–11*), and merozoite surface protein-1 (*msp1*) as an asexual parasite control. Autoradiographs are shown with the corresponding ethidium bromide-stained gel. H) Expression of gametocyte specific antigen Pfs48/45. Methanol-fixed WT, 3D7.G_def_+*Pfgdv1* (*a*) and 3D7.G_def_+HA.*Pfgdv1* (b) mature gametocyte cultures were incubated with Pfs48/45 mAb IIC5B10 (1∶250 dilution) and labeled secondary antibodies (1∶500 dilution). I) The average gametocytemia of 4 independent cultures of WT 3D7 (WT), WT 3D7 transformed with a *Pfgdv1* episomal expression plasmid (WT+HA.*Pfgdv1*) and the G_def_ (G_def_) line is graphed. The error bars represent the SEM and a significant difference from WT and G_def_ is indicated by an asterisk (p<0.05 ANOVA followed by Tukey multiple comparison test).

To study the genetic basis for the inability of the G_def_ line to make gametocytes, we used two *P. falciparum* microarrays, one with ∼7000 70-mer oligonucleotides and the other with 2.5 million 25-mer tiling oligonucleotides, to compare genomic DNA (gDNA) from the 3D7.G+ and 3D7.G_def_ lines [16], [25], [26]. A single oligonucleotide, i13417_1 (MAL9: 1,379,276–1,379,345) designated with an asterisk in Fig. 2A, had a significantly lower signal when the 70-mer oligo array was hybridized with gDNA from the 3D7.G_def_ clone than with that from the 3D7.G+ clone, suggesting substitution(s) or a deletion in the 3D7.G_def_ genome sequence. Two oligonucleotides (i14759_1 and i12812_3) flanking the i13417_1 probe hybridized to gDNA from both parasite clones with similar intensities. This hybridization data defined a DNA segment of ∼30 kb on chromosome 9 that was likely deleted in the 3D7.G_def_ clone (Fig. 2A). To confirm this observation, we designed eight PCR primer pairs to amplify the region between the two positive oligonucleotides (Fig. 2A). PCR products were obtained for all eight primer-pairs using gDNA from 3D7.G+ and wild type (WT) 3D7 lines, but only primer pairs 1, 7, and 8 amplified products from 3D7.G_def_ gDNA (Fig. 2B). DNA sequencing of a PCR product spanning the 3D7.G_def_ deletion defined the breakpoints to an 18,920 bp region (1,374,582 and 1,393,502 bp) on chromosome 9. The deletion is flanked by polyA tracts of >18 bp that have been reported to be sites of frequent recombination and deletion [27].

This region of chromosome 9 was intact in a second gametocyte-producing strain (Indochina), but was absent in two other gametocyte-deficient laboratory lines derived from FCR3 and HB3 (designated FCR3.G_def_ and HB3.G_def_) (Fig. 2B). All the parasite lines retained region 1, but regions 2 to 7 were absent in both FCR3.G_def_ and HB3.G_def_ (Fig. 2B). These results were consistent with a previous observation of a loss of both gametocyte production and cytoadherence to cells expressing CD36 after a deletion at one end of chromosome 9 and showed that the size of the deletion varies between strains [28]–[31]. Although there is a frequent loss of this region in culture, it appears to be retained in field isolates consistent with a role in gametocyte production which is required for transmission in the field, but not for asexual growth in culture [30], [32].

To further investigate whether there were changes in the genome other than the chromosome 9 deletion in the 3D7 derived clones, we used a high-density tiling array (PFSANGER GeneChip) [26] to scan the parasite genome. Comparison of gDNA isolated from WT 3D7, 3D7.G+, 3D7.G_def_, and FCR3.G_def_ confirmed the deletion on chromosome 9 in the gametocyte-deficient parasites (log ratio of 3D7.G_def_ and FCR3.G_def_ over WT 3D7 signals were −5.31; p<0.01). The only other difference detected by the tiling array between both the G_def_ lines and the G+ lines was a 342 bp region in the C-terminus of a 1,032 bp subtelomeric variable open reading frame (*stevor*) (MAL13P1.7) located in the subtelomeric region of chromosome 13. *Stevors* are highly polymorphic and variations in these loci are common. The original 70-mer microarray did not detect the difference in the *stevor* gene because of the lack of probes in this region.

The 18,920 bp region deleted in 3D7.G_def_ and the ∼26,000 bp region absent in FCR3.G_def_ contained a single annotated gene (PFI1710w or *Pfge4* on the original G+/G_def_ microarray) that was re-named *Pfgdv1* because of its putative role in *Plasmodium falciparum* gametocyte development. This gene had previously been called cytoadherence-linked protein due to its association with a loss of cytoadherence following a 0.3 Mb deletion of the entire subtelomeric region of chromosome 9 [33], but later analysis of additional lab isolates demonstrated that it was upstream from the cytoadhesion locus [34]. The C-terminal half of the predicted 72-kDa protein encoded by *Pfgdv1* contains two helix-rich regions (aa 231–312 and 508–577) (Fig. S1). Homologues in *Plasmodium reichenowi, Plasmodium vivax, Plasmodium knowlesi*, and *Plasmodium gallinaceum* have similar helix-rich domains, and all are predicted to localize to the nucleus by both PSORT and PSORTII algorithms [35]–[37], although no nucleotide binding domains were detected. There is a break in synteny with the rodent genome sequences in this region and no obvious *Pfgdv1* homologues are evident.

### *Pfgdv1* complementation restores gametocyte production in a gametocyte-deficient line

To directly test whether *Pfgdv1* has a role in gametocytogenesis, plasmids containing a blasticidin (BSD) resistance gene, the complete *Pfgdv1*predicted open reading frame (bp 1–1800) and a segment of the 5^/^ flanking region (−1236 to −1 bp from the ATG) with or without a 5^/^ hemagglutinin (HA) epitope tag (pCBM.BSD.*Pfgdv1.*5^/^.ORF and pCBM.BSD.*Pfgdv1.*5^/^.HA.ORF, respectively) were used to transform the 3D7.G_def_ clone. Blasticidin-resistant parasites obtained from two independent transformations produced gametocytes in numbers comparable to the WT 3D7 strain and significantly more gametocytes than the uncomplemented G_def_ line (peak gametocytemia 1.6–3.1% for 3D7.G_def_+*Pfgdv1*; 0.75–3.9% for 3D7.G_def_+HA.*Pfgdv1*; 1.6–4.3% for WT 3D7, and 0–0.2% for 3D7.G_def_, p<0.05, ANOVA followed by Tukey's multiple comparison) (Fig. 2D–F). In contrast, gametocytogenesis was not restored by transformation of 3D7.G_def_ with a pCBM.BSD.reporter construct alone (data not shown). Southern blot analysis confirmed that the genomic deletion was still present in the complemented lines, ruling out the possibility of WT parasite contamination (Fig. 2C). DNA fragments from restriction digestion (*Bsa*B1) confirmed the presence of the complementation plasmids in the 3D7.G_def_ +*Pfgdv1* and 3D7.G_def_ +HA.*Pfgdv1* parasites (1.1 kb and 1 kb, respectively), whereas a 4.4 kb fragment from WT 3D7 and 3D7.G*+* parasites was consistent with the presence of a genomic copy of *Pfgdv1*. The complemented parasites progressed through all five morphological stages of gametocytogenesis, and gametogenesis could be induced by conditions simulating those of the mosquito midgut, 25°C, pH>8.0 and 100 µM xanthurenic acid [15]. Further, northern analysis of RNA harvested from WT, G_def_ and the complemented lines, 3D7.G_def_ *+Pfgdv1*, and 3D7.G_def_ +HA.*Pfgdv1*, demonstrated that expression of the *Pfge* genes 1–11 as well as Pfs48/45 were restored (Fig. 2G and H).

### *Pfgdv1* overexpression enhances gametocyte production in wild type 3D7 parasites

To further confirm a role for *Pfgdv1* in the modulation of gametocytogenesis, an additional copy of *Pfgdv1* was expressed in WT 3D7 parasites using the pCBM.BSD.*Pfgdv1.*5^/^.HA.ORF episomal expression construct. As shown in Fig. 2I, transformed lines expressing a second copy of *Pfgdv1* attained a higher mean gametocytemia than the parental WT 3D7 and the G_def_ line (WT, 1.4±0.36; WT+HA.*Pfgdv1*, 4.65±1.58; G_def_, 0±0; mean ± SEM, (p<0.05 ANOVA) followed by Tukey's multiple comparison test). Together, these results demonstrate that *Pfgdv1* plays a key role in *P. falciparum* gametocytogenesis.

### PfGDV1 localizes to the nuclear periphery

**Figure 3:**
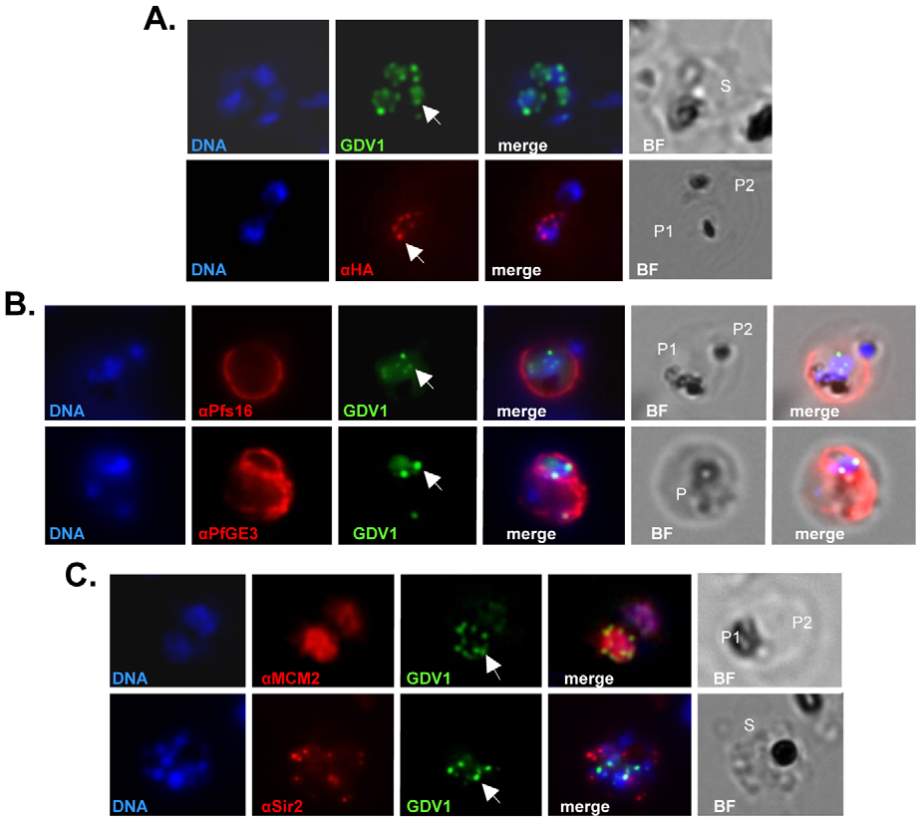
Subcellular localization of PfGDV1. Parasites transformed with GFP- or HA-tagged PfGDV1 were stained with DAPI (DNA stain) and the indicated anti-sera, and then examined by fluorescence microscopy (Zeiss Axiovert 200, 1000× magnification). Images are shown of the DAPI stain (DNA), GFP-tagged PfGDV1 epifluorescence (GDV1), and antibodies specific for HA (αHA), Pfs16 (αPfs16), PfGE3 (αPfGE3), PfMCM2 (αMCM2), and PfSir2 (αSir2). The corresponding merged and bright field (BF) images are included on the right. PfGDV1 expression is indicated with an arrow; locations of parasites in the BF image are indicated with a P for parasite or S for schizont. A) A schizont (S) (*Upper*) expressing GDV1 and a doubly infected erythrocyte (*Lower*) with one parasite (P1) expressing HA-tagged PfGDV1 (αHA) and another negative (P2) for anti-HA antibodies. B) Co-staining of parasites expressing GDV1 with early gametocytogenesis markers. A doubly infected erythrocyte (*Upper*) with one parasite (P1) positive for GDV1 and αPfs16 and the other (P2) negative for both. An erythrocyte (*Lower*) infected with a parasite (P) positive for GDV1 and αPfGE3. C) Co-localization of PfGDV1 with nuclear proteins. A doubly infected erythrocyte (*Upper*) with one parasite in the plane of the image (P1) and the other below (P2). Both P1 and P2 are positive for GDV1 and αMCM2. A schizont (S) (*Lower*) expressing GDV1 stained with αSir2.

The subcellular location of PfGDV1 was evaluated by tagging the gene with green fluorescent protein (GFP) or a HA epitope. Consistent with the nuclear localization predicted by PSORT, both tagged proteins had a punctate expression pattern around the nuclear periphery in a subpopulation (∼0.2–0.4%) of trophozoites and schizonts (Fig. 3A). Parasites expressing GFP-tagged PfGDV1 also stained with anti-sera against early gametocyte markers Pfs16 (PfGE11) and Pfg14.748 (PfGE3), suggesting that these parasites were committed to gametocytogenesis (Fig. 3B) [19], [38]. The nuclear location of PfGDV1 was further evaluated using antibodies against nuclear protein minichromosome maintenance protein 2 (MCM2) [39] or silencing information regulator 2 (Sir2) (Fig. 3C). PfMCM2, which is part of the replication origin, co-localizes with the DAPI stained nucleus in trophozoites and is internal to the PfGDV1-GFP fluorescence (Fig. 3C, upper row). PfSir2 binds to Rep20 telomere sequences and has been implicated in the silencing of *var* genes in peri-nuclear repressive centers [39], [40]. Anti-sera against PfSir2 also localized to the nuclear periphery adjacent to PfGDV1-GFP, but the two patterns did not overlap (Fig. 3C, lower row). This is the first report of a gametocyte-associated gene that has been localized to the nucleus raising the possibility that *Pfgdv1* could have a role regulating gametocyte production pathways.

### Expression profiles of *Pfgdv*1 and the *Pfge* genes identify an early gametocyte committed form (G_c_) during the transition from asexual to sexual differentiation

Development of sensitive molecular assays that distinguish committed gametocytes in the presence of asexual parasites should facilitate monitoring the transition from asexual to sexual differentiation. The identification of *Pfgdv1* and the *Pfge* genes that are selectively expressed early in gametocyte-committed (G_c_) parasites molecularly defines a sub-population of committed cells that have not yet acquired the morphological characteristics of G_II–IV_ gametocytes. To further evaluate *Pfgdv1* and the *Pfge* expression patterns as markers of gametocyte commitment, we followed emergence of the G_c_ population over multiple asexual cycles in synchronized *in vitro* cultures using RT-qPCR and compared the expression patterns with constitutively- and asexual-stage specifically expressed genes.

**Figure 4:**
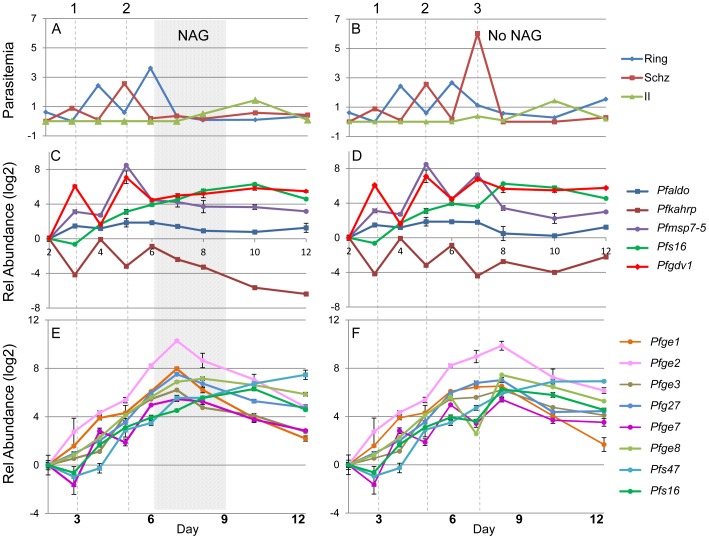
Expression profile of the *Pfge* genes through gametocytogenesis. MACS purified late stage asexual parasite cultures were set up at 6% hematocrit and sorbitol synchronized 2 hours later to remove all but the newly invaded ring stage parasites. Parasitemia was monitored by daily Giemsa-stained smear. Ring (blue), schizont stage parasitemias (red), and stage II gametocytemia (green) are plotted (A & B). The relative abundance, (log2) of the indicated gene in relation to the expression level on day 2 was calculated using the 2^−ΔΔ*C*^_T_ method [43] with seryl tRNA synthetase as the reference and plotted in C and D): *Pfaldolase* (blue), *Pfkahrp* (brown), *Pfmsp7-5* (purple), *Pfs16 (*green), *Pfgdv1* (bright red); while *Pfge1* (orange), *Pfge2* (pale pink), *Pfge3* (beige), *Pfg27* (light blue), *Pfge7* (dark pink), *Pfge8* (light green), *Pfs47*(turquoise) and *Pfs16* (green) are shown in (E and F). The 3 asexual cycles are indicated by numbers as well as gray dotted lines, and NAG treatment is indicated by the gray box. Representative data from one of three independent experiments is shown. The samples from the different time points were tested in triplicate and the average relative expression is plotted with the error bars representing the range.

Strain 3D7 *P. falciparum* gametocyte cultures were initiated with 0.1% synchronized ring stage parasites and followed daily for 2 weeks, through 3 asexual cycles (1, 2, 3, Fig. 4A and B). Asexual parasitemia peaked in 6–7 days with a subsequent decline after day 7 and a rise in Giemsa-detectable stage II gametocytes that peaked on day 10. In one set of cultures (Fig. 4A), N-acetyl-D-glucosamine (NAG) was added on day 6–8 when the ring stage parasitemia reached 4% to prevent schizont maturation and merozoite invasion as previously described [41], [42]. In the absence of NAG (Fig. 4B), ring forms observed on day 6 developed into schizonts by day 7, but did not form new ring stage parasites, causing the parasitemia to decline rapidly by day 8. Consequently, the major difference between NAG-treated and untreated cultures was the third schizont peak on day 7. In both cases the asexual parasitemia declined by day 8 and a similar number of ring stage parasites and gametocytes were produced in both conditions. These findings suggest that gametocytogenesis was induced prior to the death of the schizonts on day 7 and was not triggered directly by the conditions that cause the natural decline in asexual growth (crash). The gametocyte conversion rate was estimated to be 13.8% and 13% in NAG treated and untreated cultures, respectively, by using the daily RBC counts and Giemsa-stained culture smears to calculate the total number of gametocytes and ring stage parasites produced over the first 10 days of culture.

The expression profiles of the *Pfge* genes, the gene encoding knob associated histidine rich protein (*Pfkahrp*,PFB0100c, ring specific), and the gene encoding aldolase (PF14_0425, housekeeping gene) were determined using RT-qPCR in three independent experiments. The data was analyzed using ΔΔ cycle threshold (*C*_T)_ relative quantitation [43] and plotted as the log2 of the relative abundance with day 2 as a reference and the housekeeping gene seryl tRNA synthetase (PF07_0073) as the constitutive control (Fig. 4C–F) [44]. Peaks in *Pfgdv1* transcript levels corresponded with the peaks of schizont stage parasites, but were negatively correlated with ring stage peaks and the transcript levels of *Pfkahrp* and *Pfgeco* before day 8. Additionally with subsequent asexual cycles *Pfgdv1* RNA levels progressively increased and continued to be expressed through gametocyte formation (Fig. 4C and D; Tables 1 and 2; Fig. S2). The transcript levels of only one other *Pfge* gene, *Pfmsp7-5* also peaks in schizonts (Fig. 4C and D), whereas the rest of the *Pfge* gene transcripts gradually increased as more parasites switched to gametocytes during each asexual cycle. If we consider the expression of *Pfgdv1* as occurring very early in sexual development, then the results suggest that a subset of schizonts are already committed on day 3 prior to a rise in parasitemia. It has previously been reported that some schizonts are committed to gametocyte production [4]–[8], but no genes have been linked to this to date and the timing has not been evaluated. Transcript levels peaked on day 8 for all the *Pfge* genes in Fig. 4E and F, except *Pfs47* which continued to rise through day 12.

**Table 1:** Pearson correlation analysis of gene expression profiles in *in vitro*, NAG-treated cultures.

NAG	*kahrp*	*aldo*	*Pfgdv1*	*msp7-5*	*Pfge1*	*Pfge2*	*Pfge7*	*Pfg27*	*Pfge3*	*Pfge8*	*Pfs16*	*Pfs47*	*geco*
*kahrp*	1												
*aldo*	−0.296	1											
*Pfgdv1*	−0.788	0.598	1										
*msp7-5*	−0.326	0.456	0.608	1									
*Pfge1*	0.090	0.068	0.213	0.394	1								
*Pfge2*	−0.277	0.298	0.525	0.540	0.863	1							
*Pfge7*	−0.040	0.005	0.173	0.313	0.851	0.878	1						
*Pfg27*	−0.345	0.190	0.479	0.481	0.838	0.959	0.912	1					
*Pfge3*	−0.195	0.344	0.385	0.637	0.681	0.860	0.743	0.829	1				
*Pfge8*	−0.426	0.170	0.437	0.479	0.748	0.888	0.874	0.960	0.828	1			
*Pfs16*	−0.507	0.039	0.497	0.403	0.605	0.816	0.846	0.881	0.694	0.910	1		
*Pfs47*	−0.616	0.256	0.479	0.543	0.327	0.667	0.609	0.752	0.760	0.832	0.800	1	
*geco*	0.754	−0.486	−0.689	−0.060	0.443	0.143	0.365	0.129	0.242	0.087	−0.055	−0.108	1

The expression profiles of the relative abundance of indicated genes in relation to seryl tRNA synthetase at day 2 were compared to each other in the NAG treated (NAG) the Pearson correlation coefficient is indicated. Representative data shown is from one of 3 independent experiments.

**Table 2:** Pearson correlation analysis of gene expression profiles in *in vitro* untreated cultures.

No NAG	*kahrp*	*aldo*	*Pfgdv1*	*msp7-5*	*Pfge1*	*Pfge2*	*Pfge7*	*Pfg27*	*Pfge3*	*Pfge8*	*Pfs16*	*Pfs47*	*geco*
*kahrp*	1												
*aldo*	−0.273	1											
*Pfgdv1*	−0.863	0.476	1										
*msp7-5*	−0.423	0.361	0.581	1									
*Pfge1*	−0.244	0.199	0.371	0.626	1								
*Pfge2*	−0.445	0.253	0.622	0.567	0.874	1							
*Pfge7*	0.011	−0.035	0.232	0.321	0.782	0.853	1						
*Pfg27*	−0.379	0.237	0.556	0.569	0.870	0.975	0.862	1					
*Pfge3*	−0.315	0.204	0.435	0.527	0.829	0.891	0.820	0.940	1				
*Pfge8*	−0.182	0.053	0.359	0.289	0.639	0.800	0.825	0.781	0.855	1			
*Pfs16*	−0.339	−0.063	0.513	0.347	0.658	0.871	0.890	0.846	0.811	0.878	1		
*Pfs47*	−0.344	0.087	0.449	0.394	0.462	0.755	0.686	0.751	0.770	0.818	0.826	1	
*geco*	0.583	−0.546	−0.632	−0.025	0.317	0.076	0.416	0.193	0.273	0.171	0.119	0.060	1

The expression profiles of the relative abundance of indicated genes in relation to seryl tRNA synthetase at day 2 were compared to each other in the untreated cultures (No NAG) and the Pearson correlation coefficient is indicated. Representative data shown is from one of 3 independent experiments.

### *Pfgdv1* and *Pfge* gene expression profiles in blood samples from malaria patients

**Figure 5:**
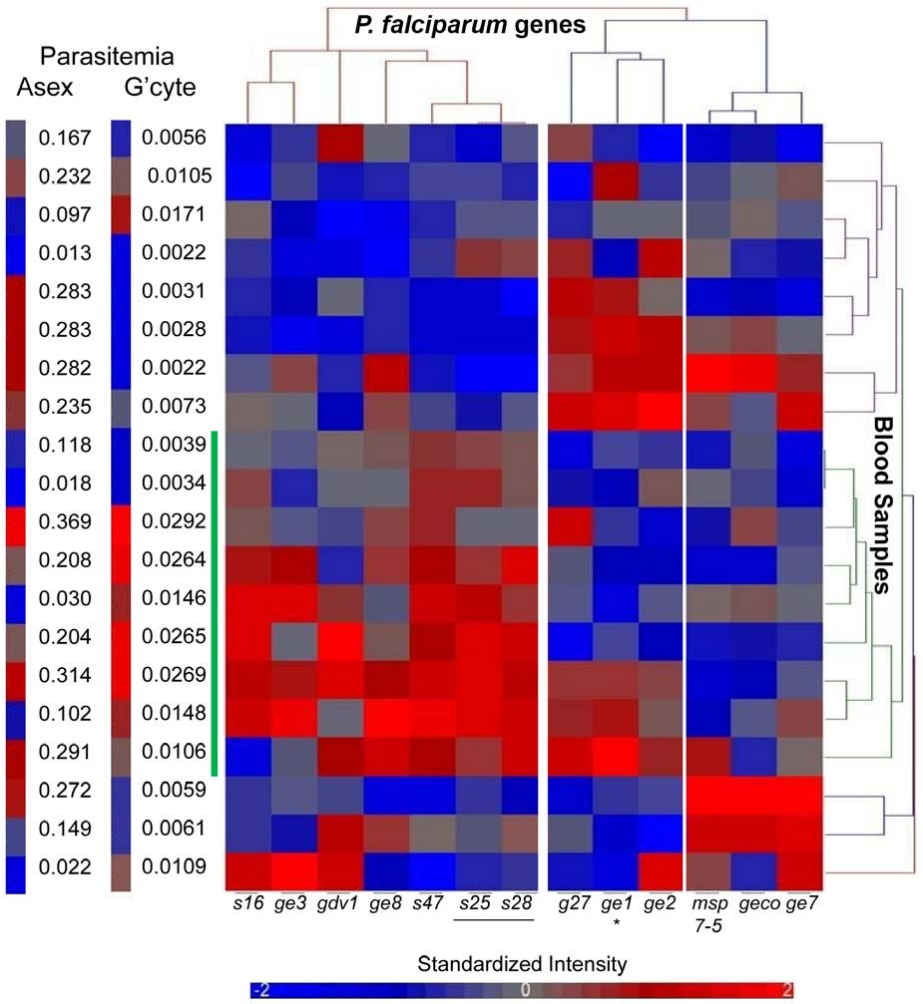
*In vivo Pfge* gene expression profiling. RNA from 20 gametocytemic patients was analyzed using a *P. falciparum* whole genome microarray. The color coded cluster analysis of the quantile normalized expression data for the *Pfge* genes with a G+/G_def_ ratio>10 is shown with mature gametocyte specific genes *Pfs25* and *Pfs28* (Underlined in gray). The gene name and standardized intensity of the color code is indicated below and the patient cluster associated with high gametocytemia is indicated on the right with a vertical green bar. The gametocytemia (G'cyte) and asexual parasitemia (Asex) of the patient samples are represented both by color code and numerically on the right.

The identification of genes that are specific for gametocyte-committed ring stage parasites provides an opportunity to evaluate whether these stages can be detected circulating in malaria patients and therefore be developed as biomarkers for gametocyte induction *in vivo*. Markers specific for early gametocytes could then be paired with mature stage V gametocytes markers, such as *Pfs25* [45], for use in future clinical studies to evaluate the parameters that affect gametocyte initiation and maturation. Since patient blood samples can contain both ring stage parasites and mature gametocytes it is important to identify genes that are expressed only in committed ring stage parasites, not mature gametocytes. *In vitro* culture is not a good model for this because it is difficult to separate immature from mature gametocytes, therefore we directly evaluated the expression profile of *Pfgdv1* and the *Pfge* genes in a microarray analysis of samples obtained from 20 patients on the Thailand Myanmar border with a range of different gametocytemias and asexual parasitemias (Fig. 5 and Table S2).

Cluster analysis of the expression profiles separated the genes into 2 major groups. One group included *Pfs16*, *Pfs47*, *Pfge8*, *Pfge3* and *Pfgdv1*, as well as late gametocyte markers *Pfs25* and *Pfs28* and had higher expression levels in patient samples with higher gametocytemias (p = 0.0185 Mann Whitney) limiting their usefulness as biomarkers for gametocyte committed rings in field samples. The other group could be subdivided into 2 clusters, one containing *Pfgeco*, *Pfmsp7-5* and *Pfge7* and another containing *Pfge1*, *Pfge2* and *Pfg27*. Significantly, the expression profile of *Pfge1* correlated with asexual parasitemia even when corrected for false discovery (FDR) and controlled for gametocytemia (ANCOVA analysis, correlation = 0.667, p = 0.002, FDR q-value = 0.010) (Fig. 5). Given the previous data establishing the specificity of these genes for early gametocytes [19] and their *in vitro* expression pattern (Fig. 4E and F), these results suggest that they are good candidates for further evaluation as markers for different stages of gametocyte commitment in the field. The close correlation between the expression of *Pfge1* and asexual parasitemia is also consistent with the ongoing induction of gametocytes during asexual growth that was observed *in vitro* and should be analyzed in future field studies.

## Discussion

The present study identified a *P. falciparum* gene, *Pfgdv1*, that plays a role in early gametocytogenesis. A set of downstream genes involved in early events in sexual development were also identified, and together these permitted analysis of gametocyte induction in synchronized cultures and patient samples. The role of *Pfgdv1* in gametocytogenesis was demonstrated through four lines of evidence, 1) the association of a genomic deletion containing *Pfgdv1* in 3 spontaneously occurring gametocyte-deficient lines; 2) restoration of gametocyte production following complementation of a 3D7 gametocyte-deficient line with WT *Pfgdv1*; 3) enhancement of gametocyte production in WT parasites following overexpression of *Pfgdv1* using an episomal copy of the gene and 4) a peri-nuclear location of epitope tagged-PfGDV1 in a subpopulation of schizonts, as well as early pre stage I gametocytes co-expressing *Pfs16* and *Pfge3*. Molecular complementation is an important control for inadvertent mutations, and this is the first report of restoration of gametocyte production by complementation of *P. falciparum*. Overexpression studies not only provide evidence that increases in *Pfgdv1* activity modulate gametocyte production in a dynamic fashion, but may also provide a molecular tool to augment the production of gametocytes to facilitate the development of transmission-blocking reagents [46]. Previous work demonstrated that episomal expression of a neighboring gene *Pfgig* (PFI1720w) increased *Pfs16* expression in a non-gametocyte producing line, but did not restore gametocyte production [29]. *Pfgig* was intact in the G_def_ line used in this study and expression was not significantly affected by the loss of *Pfgdv1*; however, it is possible that it could act in conjunction with *Pfgdv1* to stimulate gametocytogenesis.

The presence of a *Pfgdv1* homologue in the primate malarias *P. vivax* and *P. knowlesi*, as well as the avian malaria *P. gallinaceum*, suggests a shared role in early gametocyte formation since later steps in gametocyte development, such as changes in morphology and the time course of maturation, are quite different between these species [47]–[49]. The apparent absence of a *Pfgdv1* homologue in the rodent malarias is intriguing, given the currently accepted phylogeny that separates avian and mammalian *Plasmodium* [47]–[49]. If the gene is not found in the rodent *Plasmodium* genomes, it is possible that gametocyte induction is distinct in the rodent *Plasmodium* or that another gene with a similar structure, but a unique amino acid sequence is involved. In contrast to *Pfgdv1*, eight of the *Pfge* genes are unique to the *Laverania* subspecies of *P. falciparum*, which includes *P. falciparum* and *P. reichenowi* and is characterized by morphologically distinct gametocytes that sequester during a prolonged 10–12 day gametocyte maturation period [47]. Seven of these *Laverania*-specific *Pfge* genes are predicted to be secreted and exported proteins and could be involved in adaptations to the RBC environment that are required for sequestration and survival over 10–15 days in an immune competent host [19], [23], [24], [50]–[54].

The lack of expression of the *Pfge* genes in parasites with defective *Pfgdv1* is consistent with *Pfgdv1* playing a key upstream role in early gametocytogenesis or commitment to sexual development. The transcription profile of *Pfgdv1* is consistent with the observed pattern of PfGDV1 protein expression in a subpopulation of schizonts and continued expression in early G_c_ parasites. It is tempting to speculate that the peri-nuclear location of PfGDV1 in gametocyte-committed schizonts could indicate a role in the regulation of gametocytogenesis [55], [56]. Lack of homology with known transcription factors or nucleotide-binding proteins suggests that instead of a direct role in gene expression, PfGDV1 could be part of a regulatory complex. Developmental switches often include a number of scaffold and regulatory proteins, which could serve as a model for *Pfgdv1*. Examples in yeast include Far1 that binds Cdc28- Cln2 kinase causing cell cycle arrest [57] and Rmf1 that tethers histone deacetylase Hst1 and the DNA-binding protein Sum1 [58]. As the first protein found to localize to the nucleus during sexual differentiation, PfGDV1 provides a starting point for identifying the additional functional components of the regulatory cascade in the sexual development of *P. falciparum*.

**Figure 6:**
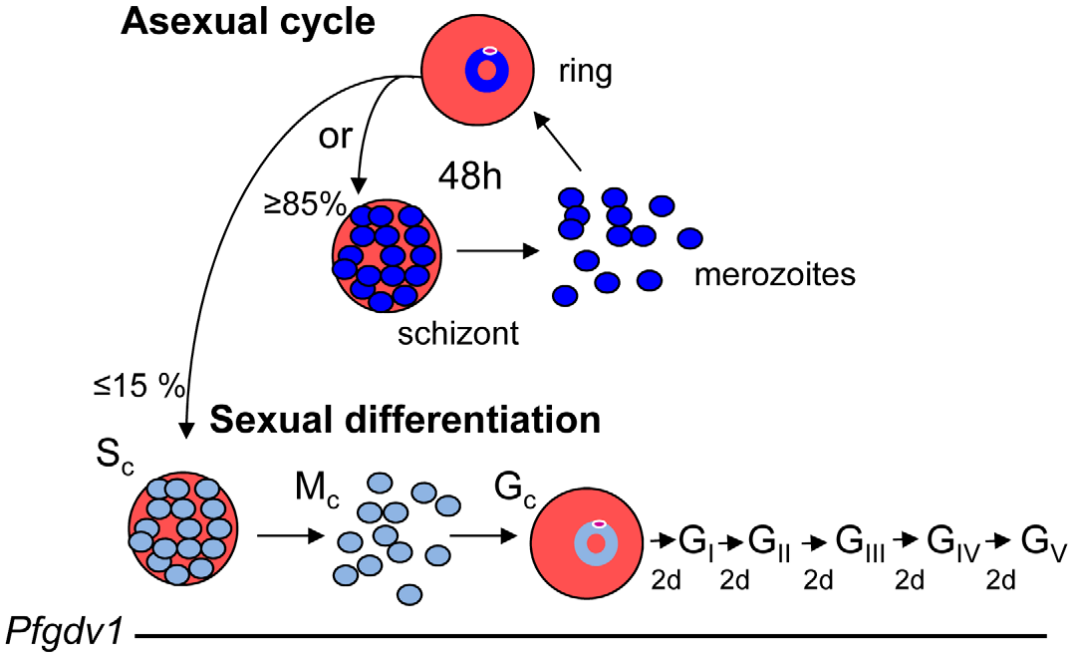
Model for continuous gametocytogenesis. During each round of the asexual cycle, a proportion of the schizonts (S_c_, light blue) produced are committed to producing merozoites (M_c_, light blue) that will differentiate into gametocytes (G_c_, G_I–V_) after invading a RBC. The expression profile for *Pfgdv1* is indicated by a line under the corresponding stage.

The expression of *Pfgdv1* and the *Pfge* genes through multiple rounds of asexual growth of strain 3D7 parasites and continued expression for 24 hours after the clearance of asexual parasites is in marked contradiction to the hypothesis that gametocyte differentiation is induced by the same stress imposed by high density culture that inhibits further asexual growth. Additionally, blocking the development of high parasitemia on day 7 using NAG did not affect gametocyte production. However, other parasite strains will have to be tested to determine if this is a general characteristic. These data suggest that in strain 3D7 parasites, a subpopulation of parasites convert to gametocytogenesis during each asexual cycle, instead of a model where the majority of parasites differentiate in response to a onetime stimulation event as seen in yeast after exposure to stress-induced mating pheromone, a quorum response in bacteria to high cell levels, or induction factor stimulation of trypanosomes [13], [59]–[62]. The ongoing conversion of a subpopulation of intraerythrocytic parasites to sexual differentiation during each asexual cycle fits well with the pattern of gametocyte production observed in neurosyphilis patients receiving malaria therapy [63], [64]. During the long course of malaria therapy, the rise of circulating stage V gametocytes follows the same pattern as the increase in asexual parasitemia, but is delayed by the 10 days required for gametocyte maturation and plateaus at ∼10% of the maximal asexual parasitemia. These findings suggest that during each asexual cycle ∼10% of the parasites are committed to gametocytogenesis (Fig. 6). This ratio is similar to the gametocyte conversion rate reported for *P. vivax* [65], and the ratio of ring stage parasites to stage II gametocytes observed in our *in vitro* cultures (13.35%±0.175, mean ± SEM), suggesting a consistent sexual induction rate *in vitro* and *in vivo*. A model of continuous gametocyte induction during each asexual erythrocytic cycle is also supported by the significant correlation found between *Pfge1* expression levels with asexual parasitemia in patients in the current study and previous reports that gametocytes can be detected in >90% of malaria patients when analyzed by quantitative-nucleic acid sequence-based amplification [63], [64]. The continuous differentiation of a subpopulation of progeny from a reservoir of self-renewing stem cells seen in sperm formation or hematopoiesis may be a better model for gametocytogenesis than sexual differentiation in yeast [66], [67]. Such a strategy of ongoing gametocyte production could facilitate transmission by providing a continuous source of infectious gametocytes whenever a mosquito takes a blood meal.

Concurrent gametocyte induction and asexual growth does not rule out modulation of gametocyte production by environmental conditions or host/parasite genetics. As demonstrated here, gametocyte production was enhanced by *Pfgdv1* overexpression in WT parasites and other studies show that different parasite lines produce different numbers of gametocytes and gametocyte yields vary in different batches of *in vitro* culture [61], [68], [69]. Again, this type of modulation could be similar to hematopoiesis where the cytokine milieu influences the number and type of blood cells produced by altering maturation without stimulating all the stem cells to differentiate, thus maintaining an asexually replicating stem cell stock [67]. In humans, variation in gametocyte levels have been associated with anemia (<30 hematocrit), duration of illness and drug treatment, but whether the changes were due to differences in gametocyte induction or maturation was not evaluated [70]–[73]. The identification of these *Pfge* genes provides new tools to evaluate gametocytogenesis directly in humans. If confirmed, the continuous commitment to gametocytogenesis suggests a long transmission window during human infections and reinforces the need to include these essential stages of the parasite life cycle in malaria control efforts. It also suggests the importance of developing methods that provide widespread, long lasting protection, such as vaccines or drug combinations that are effective against all erythrocytic stages.

## Materials and Methods

### Ethics statement

All research involving human subjects was reviewed and approved by the Institutional Ethics Committee of the Thai Ministry of Public Health and the Human Subjects Research Review Board of the United States Army and have been conducted according to the principles expressed in the Declaration of Helsinki. Written informed consent was provided by study participants and/or their legal guardians.

### *P. falciparum* parasite strains

*P. falciparum* strain 3D7 was provided by D. Keister (National Institute of Allergy and Infectious Diseases). *P. falciparum* parasites FCR3.G_def_, HB3.G_def_ and Indochina were provided by M. Klemba (Virginia Institute of Technology). WT 3D7 was transformed with pDT.Tg23.230-D1.356 (pD1.356) to study the role of gamete surface antigen *Pfs230*, and two independently transformed, pyrimethamine-resistant lines were obtained as described [14]. One clonal line produced gametocytes (3D7.G+), whereas the other was gametocyte-deficient (3D7.G_def_), neither expressed full length Pfs230. Parasites were synchronized using sorbitol treatment or MACS columns (Miltenyi Biotec, Auburn, CA), and gametocyte cultures were set up at 0.1% ring stage parasitemia [19], [74]. Parasite stages were quantitated using Giemsa-stained culture smears to determine parasitemia and total red blood cell counts.

### DNA and RNA isolation and analysis

Parasite gDNA was isolated using the Wizard gDNA Purification kit (Promega, Madison, WI), and RNA was isolated from parasites at the indicated developmental stage using TRIzol (Life Technologies, Grand Island, NY) as described [19]. For Southern and northern analyses, gDNA digested with the indicated restriction enzymes (New England Biolabs, Ipswich, MA) or RNA samples were size-fractionated and transferred to Nytran membrane (Schleicher and Schuell, Keene, NH), respectively. The membranes were incubated with randomly primed α^32^P-dTTP-labeled probe in 1× DIG hybridization buffer (Roche, Indianapolis, IN) at 65°C overnight, washed twice in 0.2× SSPE (30 mM NaCl, 2 mM NaH_2_PO_4_, 0.2 mM EDTA) and 0.5% Sodium dodecyl sulfate for 30 m at 65°C, and visualized by autoradiography.

### Microarray analysis

#### Parasite gDNA

Two different platforms were used to analyze parasite gDNA: A) the 70-mer oligonucleotide array designed by the DeRisi laboratory (Operon Technologies, Alameda, CA) [16] and B) the PFSANGER GeneChip (Affymetrix, Inc., Santa Clara, CA) designed at the Sanger Center [26]. For the 70-mer oligonucleotide array, fluorescently labeled probes were generated from parasite gDNA by random prime labeling with Cy5- or Cy3-dUTP, in a 50 µl reaction containing 50 mM Tris, pH 6.8, 5 mM MgCl_2_, 100 µM EDTA, 10 mM 2-mercaptoethanol, 300 µg/ml random hexamer, 120 µM each dATP, dGTP, and dCTP, 60 µM dTTP, 60 µM Cy5-dUTP or Cy3-dUTP, and 50 U Klenow fragment. The mixture was incubated at 37°C for 2 h and then stopped by increasing EDTA to 50 mM. Labeled probes were purified by filtering through six successive washes in TE pH 8.0 (10 K MWCO Vivaspin 500, Vivascience AG, Hanover, Germany) and used in the MAUI hybridization system (BioMicro Systems, Salt Lake City, UT). Array hybridization was performed by the Genomic Technologies Section (National Institutes of Health, Bethesda, MD) with a GenePix 4000B array scanner (Axon Instruments, Union City, CA). The microarray signals from each array spot were normalized so that the median Cy5∶Cy3 ratio was set as 1.0. Collected data were analyzed with GenePix Pro 4.0 (Axon Instruments) and web-based microarray tools (mAdb) developed by the Bioinformatics and Molecular Analysis Section, Center for Information Technology, NIH (https://madb.niaid.nih.gov). For the PFSANGER GeneChip, parasite gDNA was fluorescently labeled as described [18], [26] and assayed at the microarray facility of the Laboratory of Immunopathogenesis and Bioinformatics, SAIC-Frederick, Inc. (Frederick, MD) as previously described [26]. The scanned-image CEL files were processed using a R/Bioconductor package and the robust multichip multi-array average algorithm [26].

#### Parasite RNA

RNA isolated from synchronized 3D7.G+ and 3D7.G_def_ clones was used to generate fluorescently labeled cDNA as previously described [19] (Table S1) and assayed at NIAID Microarray Research Facility, National Institutes of Health (NIH), Bethesda using the70-mer oligonucleotide array designed by the DeRisi laboratory (Operon Technologies) [16] as previously described [19].

#### Patient samples

Patients presenting with *P. falciparum* at clinics in the Mae Sod district of Tak province in northwestern Thailand were asked if they would be willing to donate blood to study malaria transmission. After completing the informed consent, a venous blood sample was obtained and the plasma was removed by centrifugation and the remaining cell pellet was stored frozen at −80°C. The protocols used to obtain the human samples were reviewed and approved by the Institutional Ethics Committee of the Thai Ministry of Public Health and the Human Subjects Research Review Board of the United States Army.

#### Whole genome expression profiling

DNA microarrays were long-oligo on glass from Agilent Technologies, Santa Clara, CA designed with custom content. The 44 K spots on each array included probes for 5,254 *P. falciparum* 3D7 transcript sequences (downloaded from NCBI RefSeq, December 2009). Probes (60-mer) were designed using Agilent eArray software (http://earray.chem.agilent.com/earray/) requesting one probe per target sequence using “best probe” methodology. The array also included probes for 565 human housekeeping-gene transcripts, three spot-replicates each.

Total RNA was isolated from the frozen blood cell pellet using TRIzol (Life Techonolgies, Grand Island, NY) and purified with the RNeasy Micro kit (Qiagen, Valencia, CA) and fluorescent labeled using the QuickAmp kit (Agilent). Cy dye labeled cDNA from infectious and non-infectious patient samples, paired based on similar asexual and gametocyte parasitemias, were co-hybridized on the same array. Each pair was labeled and hybridized again for dye-flip replicates. Hybridizations were incubated for 17 hr at 65°C on a TECAN HS Pro hybridization station using Agilent hybridization buffers, dried under nitrogen and scanned using a Agilent Microarray Scanner (G2505C) in an ozone-free environment at 5 micron resolution using the extended dynamic range (XDR) mode at a 100∶10 ratio. Image analysis was performed using Feature Extraction software (version 10.7.3.1) with protocol GE2_107_Sep09. Background detrend and multiplicative detrend signals were used to account for any spatial effect on the array while no other background subtraction was applied. Dye effect was removed by using linear global normalization followed by non-linear lowess normalization. The processed signals were then subjected to quantile normalization and log transformation in Partek Genomics Suite 6.5 (Partek Inc., St. Louis, MO). Signals from dye swapping samples were then averaged using geometric mean. This provided intensity values for each transcript in individual samples which were comparable across arrays. This intensity-based analysis of dual color microarray data has been shown to give reproducible results and increase sensitivity compared to ratio-based analysis [75]. Unsupervised hierarchical cluster of samples based on a given list of genes was performed in Partek Genomics Suite 6.5 with sample distance determined by Euclidean distance and cluster distance determined by average linkages between all pairs of samples. Genes with expression profiles that correlated with either asexual parasitemia or gametocytemia were assessed by statistical analysis of covariance (ANC OVA). False discovery rate (FDR) was calculated to control for multiple comparisons using the q-value method (FDR q-value) [76].

### Chromosome 9 deletion mapping

PCR primers (Table S3) spaced at ∼2-kb intervals spanning the region on chromosome 9 between oligonucleotides i14759_1 and i12812_3 (DeRisi laboratory) were used to amplify gDNA isolated from Indochina, 3D7.G_def_, FCR3.G_def_ and HB3.G_def_ parasite lines.

### Parasite transformations

*P. falciparum* parasites were transformed using established protocols as previously described [14], [77]. The complementation and reporter-tagging plasmids used are described below and the primers used for the amplifications are listed in Table S3.

#### Complementation

The 5′ flanking region, bp −1236 to −1 from the start codon of *Pfgdv1*, was amplified using primers *Pfgdv1*.5′FR.-1236.forward and *Pfgdv1*.5′FR.1.reverse and standard PCR conditions, 35 cycles of 94°C 30 s, 45°C 30 s, 60°C 6 m. The PCR product was digested with *Sac*II and *Xba*I and ligated into pCBM.BSD [78]. Once the sequence of the resulting plasmid pCBM.BSD.*Pfgdv1*.5′ was confirmed it was digested with *Xba*I and ligated to a *Xba*I-digested PCR product encoding *Pfgdv1* (bp 1–1800, chr9:1,377,942–1,379,741) generated using primers *Pfgdv1*.1.forward.*Xba*I and *Pfgdv1*.1800.reverse.*Xba*I.

#### Pfgdv1 reporter tagging

*Pfgdv1* was tagged with either hemagglutinin (HA) or green fluorescent protein (GFP) to investigate the localization of the protein. Synthetic oligonucleotides encoding the HA epitope tag flanked 5′ by a partial *Xba*I site and 3′ by *Spe*I and a partial *Bam*HI site were phosphorylated with T4 Polynucleotide kinase (New England Biolabs), incubated at 65°C for 10 m in Sequenase buffer (Life Technologies) and annealed by step-wise cooling (42°C for 5 m, 37°C for 5 m, 25°C for 10 m). The annealed oligonucleotides were ligated into *Xba*I and *Bam*HI digested pCBM.BSD.*Pfgdv1*.5′. After the insertion was confirmed by sequencing, a PCR product encoding the *Pfgdv1* coding region (bp 1 to 1800) flanked by *Spe*I and *Bam*HI sites generated using primers *Pfgdv1*.1.forward.*Spe*I and *Pfgdv1*.1800.reverse.*Bam*HI was inserted into *Spe*I and *Bam*HI digested pCBM.BSD.*Pfgdv1*.5′ HA to produce pCBM.BSD.*Pfgdv1*.5′HA.ORF. The entire *Pfgdv1*.5′ HA.ORF fragment was removed by digestion with *Sal*I and *Bam*HI and ligated into *Sal*I and *Bam*HI-digested pDT.Tg23 [79] to generate pDT.Tg23.*Pfgdv1*.5′ HA.ORF.

To generate GFP-tagged PfGDV1, the HA epitope and *Pfgdv1* ORF in pDT.Tg23.*Pfgdv1*.5′ HA.ORF were replaced by a PCR product encoding *Pfgdv1* (1–1797 bp) flanked by *Xba*I and *Spe*I sites generated using primers *Pfgdv1*.1.forward.*Xba*I and *Pfgdv1*.1797.reverse.*Spe*I to produce pDT.Tg23.*Pfgdv1*.5′.ORF. A *Spe*I and *Bam*HI-digested PCR product containing the GFP coding sequence [80] was generated using primers GFP.*Spe*I.forward and GFP.stop.*Bam*HI.reverse and inserted into *Spe*I and *Bam*HI-digested pDT.Tg23.*Pfgdv1*.5′.ORF to yield pDT.Tg23.22.*Pfgdv1*.5′.ORF.GFP.

### Indirect immunofluorescence assay

After adherence to poly-L-lysine-coated slides, parasites were fixed in 1% formaldehyde and permeabilized with 0.1% saponin, then incubated with primary and secondary antibodies as described [19]. Anti-sera specific for Pfs16 (1∶1000) [19], PfGE3 (1∶200) [19], or PfMCM2 (1∶500) [39] were used with Alexa Fluor 594-labeled anti-mouse IgG (Life Technologies) (1∶250), and anti-serum specific for PfSir2 (1∶100) [81] was used with Alexa Fluor 594-labeled anti-rabbit IgG (Life Technologies) (1∶100).

### Reverse transcriptase-quantitative polymerase chain reaction

RNA was prepared from TRIzol extracts of parasite cultures using the RNeasy Micro kit (Qiagen) according to the manufacturer's instructions. Samples (1.5 ml) were collected daily from the initial 5% hematocrit cultures and after the cultures were diluted to 2.5% hematocrit the samples were increased to 3 ml. To maintain the cell/media ratio in the culture, the amount of media used to feed the culture each day was decreased according to the sample volume removed. In addition to DNase treatment during RNA isolation on the micro columns, purified RNA (50 ng) was treated with gDNA wipeout buffer before conversion to cDNA using Quantitect reverse transcriptase (Qiagen). Reverse transcriptase minus controls were used to confirm the absence of genomic DNA and the cDNA was used as a template for RT-qPCR (StepOnePlus, Applied Biosystems) with the indicated primers (Table S3) and SYBR Green PCR Master Mix (Applied Biosystems) using the following conditions, 5 minute activation at 95°C, 40 cycles of 10 secs at 95°C and 30 secs at 60°C. All samples were run in triplicate and tested for both the gene of interest and the control constitutive gene, seryl tRNA synthetase (PF07_0073), on the same plate. The results were analyzed using StepOne plus V 2.1 Software (Applied Biosystems) and the Δ*C*_T_ values determined by subtracting the mean threshold cycle (*C*_T_) values of the target gene and seryl tRNA synthetase. To evaluate the time course, the relative quantity (2^−ΔΔ*C*^_T_) was calculated for each day in reference to the Δ*C*_T_ on day 2 [43]. The efficiency of the primers was tested by serial dilution and ranged from 88–95%. The experiment was repeated 3 times and the average inter-experimental Pearson correlation coefficient for the expression patterns of the *Pfge* genes evaluated was 0.802±0.066 (mean ± SEM).

### Gene accession numbers

*Pfgdv1*, PFI1710w; *Pfge1*, PF14_0744; *Pfge2*, PF14_0745; *Pfge3*, PF14_0748; *Pfg27*, PF13_0011; *Pfgeco*, PFL2550w; *Pfge7*, PF14_0736; *Pfge8*, PF14_0735; *msp7-5*, PF13_0196; *Pfs47*, PF13_0248; *Pfs16*, PFD0310w; PF14_0588; PF14_0290; PF08_0002; PF14_0708; PFC0680w; *Pfmdv1/Pfeg3*, PFL0795c; PF11_0038; PF08_0033; PF14_0010; Pfs230, PFB0405w; MCM2, PF14_0177; *Sir2A*, PF13_015; *kahrp*, PFB0100c; *aldolase*, PF14_0425; *seryl tRNA synthetase*, PF07_0073; *Pfs25*, PF10_0303; *Pfs28*, PF10_0302.

## Supporting Information

Figure S1 **Alignment of PfGDV1 with homologs in other** ***Plasmodium*** **species.** The predicted amino acid sequence of *Pfgdv*1 (PFI1710w) was aligned with homologs identified by BLAST searches of translated sequences on www.PlasmoDB.org, www.ncbi.nlm.nih.gov, and www.sanger.ac.uk/pathogens/malaria using NPS@: Network Protein Sequence Analysis at www.expasy.org. Shown is the CLUSTALW alignment of PfGDV1 (PFI1710w) with *Plasmodium reichenowi* (Pr332h11.p1k), *Plasmodium vivax* (PVX_086950), *Plasmodium knowlesi* (PK_073340), and *Plasmodium gallinaceum* (Pgal0953d04.p1k) (37). The PfGDV1 sequence and exact matches with other species are highlighted in yellow. Identical amino acids (aa) are shown in red, highly similar aa are green and weakly similar aa are blue. The two helix-rich regions predicted by the GOR4 algorithm are underlined.
(DOC)

Figure S2 **Expression profile of** ***Pfge6*** **(** ***Pfgeco*** **) through gametocytogenesis.** MACS purified late stage parasite cultures were set up at 6% hematocrit and sorbitol synchronized 2 hours later to remove all but the newly invaded ring stage parasites. The relative abundance of the RNA corresponding to the indicated gene in relation to the seryl tRNA synthetase ratio on day two is graphed: *Pfkahrp* (brown), *Pfge6*-*geco* (olive green), *Pfge11-Pfs16* (green) *and Pfgdv1* (bright red). The 3 asexual cycles are indicated by numbers as well as gray dotted lines and NAG treatment is indicated by the gray box. Data from one of three independent experiments is shown. The samples from the different time points were tested in triplicate and the average relative expression is plotted with the error bars representing the range.
(PDF)

Table S1 **Genes differentially expressed in the 3D7.G+ and 3D7.G_def_ clones.**
(DOC)

Table S2 **Clinical and hematological characteristics of the patient cohort.**
(DOCX)

Table S3 **Oligonucleotide primers.**
(DOCX)

## References

[1] Organization WHO (2010) World malaria report 2010.

[2] Kuehn A, Pradel G (2010) The coming-out of malaria gametocytes. J Biomed Biotechnol 2010: 976827. 20111746 10.1155/2010/976827 PMC2810480

[3] Okell LC, Drakeley CJ, Ghani AC, Bousema T, Sutherland CJ (2008) Reduction of transmission from malaria patients by artemisinin combination therapies: a pooled analysis of six randomized trials. Malar J 7: 125. 18613962 10.1186/1475-2875-7-125 PMC2491628

[4] Bruce MC, Alano P, Duthie S, Carter R (1990) Commitment of the malaria parasite *Plasmodium falciparum* to sexual and asexual development. Parasitology 100 Pt 2: 191–200. 2189114 10.1017/s0031182000061199

[5] Inselburg J (1983) Gametocyte formation by the progeny of single *Plasmodium falciparum* schizonts. J Parasitol 69: 584–591. 6355424

[6] Silvestrini F, Alano P, Williams JL (2000) Commitment to the production of male and female gametocytes in the human malaria parasite *Plasmodium falciparum* . Parasitology 121 Pt 5: 465–471. 11128797 10.1017/s0031182099006691

[7] Smith TG, Lourenco P, Carter R, Walliker D, Ranford-Cartwright LC (2000) Commitment to sexual differentiation in the human malaria parasite, *Plasmodium falciparum* . Parasitology 121 (Pt 2) 127–133. 11085232 10.1017/s0031182099006265

[8] Hawking F, Wilson ME, Gammage K (1971) Evidence for cyclic development and short-lived maturity in the gametocytes of *Plasmodium falciparum* . Trans R Soc Trop Med Hyg 65: 549–559. 5003557 10.1016/0035-9203(71)90036-8

[9] Lobo CA, Kumar K (1998) Sexual differentiation and development in the malaria parasite. Parasitol Today 14: 146–150. 17040732 10.1016/s0169-4758(97)01210-6

[10] Alano P (2007) *Plasmodium falciparum* gametocytes: still many secrets of a hidden life. Mol Microbiol 66: 291–302. 17784927 10.1111/j.1365-2958.2007.05904.x

[11] Liu Z, Miao J, Cui L (2011) Gametocytogenesis in malaria parasite: commitment, development and regulation. Future Microbiol 6: 1351–1369. 22082293 10.2217/fmb.11.108 PMC5711484

[12] Heitman J (2010) Evolution of eukaryotic microbial pathogens via covert sexual reproduction. Cell Host Microbe 8: 86–99. 20638645 10.1016/j.chom.2010.06.011 PMC2916653

[13] Baker DA (2010) Malaria gametocytogenesis. Mol Biochem Parasitol 172: 57–65. 20381542 10.1016/j.molbiopara.2010.03.019 PMC2880792

[14] Eksi S, Stump A, Fanning SL, Shenouda MI, Fujioka H, et al (2002) Targeting and sequestration of truncated Pfs230 in an intraerythrocytic compartment during *Plasmodium falciparum* gametocytogenesis. Mol Microbiol 44: 1507–1516. 12067340 10.1046/j.1365-2958.2002.02986.x

[15] Eksi S, Czesny B, van Gemert GJ, Sauerwein RW, Eling W, et al (2006) Malaria transmission-blocking antigen, Pfs230, mediates human red blood cell binding to exflagellating male parasites and oocyst production. Mol Microbiol 61: 991–998. 16879650 10.1111/j.1365-2958.2006.05284.x

[16] Bozdech Z, Llinas M, Pulliam BL, Wong ED, Zhu J, et al (2003) The Transcriptome of the Intraerythrocytic Developmental Cycle of *Plasmodium falciparum* . PLoS Biol 1: E5 Epub 2003 Aug 2018. 12929205 10.1371/journal.pbio.0000005 PMC176545

[17] Young JA, Fivelman QL, Blair PL, de la Vega P, Le Roch KG, et al (2005) The *Plasmodium falciparum* sexual development transcriptome: a microarray analysis using ontology-based pattern identification. Mol Biochem Parasitol 143: 67–79. 16005087 10.1016/j.molbiopara.2005.05.007

[18] Furuya T, Mu J, Hayton K, Liu A, Duan J, et al (2005) Disruption of a *Plasmodium falciparum* gene linked to male sexual development causes early arrest in gametocytogenesis. Proc Natl Acad Sci U S A 102: 16813–16818. 16275909 10.1073/pnas.0501858102 PMC1277966

[19] Eksi S, Haile Y, Furuya T, Ma L, Su X, et al (2005) Identification of a subtelomeric gene family expressed during the asexual-sexual stage transition in *Plasmodium falciparum* . Mol Biochem Parasitol 143: 90–99. 15996767 10.1016/j.molbiopara.2005.05.010

[20] Bruce MC, Carter RN, Nakamura K, Aikawa M, Carter R (1994) Cellular location and temporal expression of the *Plasmodium falciparum* sexual stage antigen Pfs16. Mol Biochem Parasitol 65: 11–22. 7935618 10.1016/0166-6851(94)90111-2

[21] Alano P, Premawansa S, Bruce MC, Carter R (1991) A stage specific gene expressed at the onset of gametocytogenesis in *Plasmodium falciparum* . Mol Biochem Parasitol 46: 81–88. 1852178 10.1016/0166-6851(91)90201-g

[22] van Schaijk BC, van Dijk MR, van de Vegte-Bolmer M, van Gemert GJ, van Dooren MW, et al (2006) Pfs47, paralog of the male fertility factor Pfs48/45, is a female specific surface protein in *Plasmodium falciparum* . Mol Biochem Parasitol 149: 216–222. 16824624 10.1016/j.molbiopara.2006.05.015

[23] Morahan BJ, Strobel C, Hasan U, Czesny B, Mantel PY, et al (2011) Functional Analysis of the Exported Type IV HSP40 Protein PfGECO in *Plasmodium falciparum* Gametocytes. Eukaryot Cell 10: 1492–1503. 21965515 10.1128/EC.05155-11 PMC3209067

[24] Silvestrini F, Lasonder E, Olivieri A, Camarda G, van Schaijk B, et al (2010) Protein export marks the early phase of gametocytogenesis of the human malaria parasite *Plasmodium falciparum* . Mol Cell Proteomics 9: 1437–1448. 20332084 10.1074/mcp.M900479-MCP200 PMC2938084

[25] Behr MA, Wilson MA, Gill WP, Salamon H, Schoolnik GK, et al (1999) Comparative genomics of BCG vaccines by whole-genome DNA microarray. Science 284: 1520–1523. 10348738 10.1126/science.284.5419.1520

[26] Jiang H, Yi M, Mu J, Zhang L, Ivens A, et al (2008) Detection of genome-wide polymorphisms in the AT-rich *Plasmodium falciparum* genome using a high-density microarray. BMC Genomics 9: 398. 18724869 10.1186/1471-2164-9-398 PMC2543026

[27] Nair S, Nash D, Sudimack D, Jaidee A, Barends M, et al (2007) Recurrent gene amplification and soft selective sweeps during evolution of multidrug resistance in malaria parasites. Mol Biol Evol 24: 562–573. 17124182 10.1093/molbev/msl185

[28] Alano P, Roca L, Smith D, Read D, Carter R, et al (1995) *Plasmodium falciparum*: parasites defective in early stages of gametocytogenesis. Exp Parasitol 81: 227–235. 7556565 10.1006/expr.1995.1112

[29] Kemp DJ, Thompson J, Barnes DA, Triglia T, Karamalis F, et al (1992) A chromosome 9 deletion in *Plasmodium falciparum* results in loss of cytoadherence. Mem Inst Oswaldo Cruz 87 Suppl 3: 85–89. 1343730 10.1590/s0074-02761992000700011

[30] Day KP, Karamalis F, Thompson J, Barnes DA, Peterson C, et al (1993) Genes necessary for expression of a virulence determinant and for transmission of *Plasmodium falciparum* are located on a 0.3-megabase region of chromosome 9. Proc Natl Acad Sci U S A 90: 8292–8296. 8367496 10.1073/pnas.90.17.8292 PMC47335

[31] Gardiner DL, Dixon MW, Spielmann T, Skinner-Adams TS, Hawthorne PL, et al (2005) Implication of a *Plasmodium falciparum* gene in the switch between asexual reproduction and gametocytogenesis. Mol Biochem Parasitol 140: 153–160. 15760655 10.1016/j.molbiopara.2004.12.010

[32] Bourke PF, Holt DC, Sutherland CJ, Currie B, Kemp DJ (1996) Positional cloning of a sequence from the breakpoint of chromosome 9 commonly associated with the loss of cytoadherence. Ann Trop Med Parasitol 90: 353–357. 10.1080/00034983.1996.11813063 8944078

[33] Trenholme KR, Gardiner DL, Holt DC, Thomas EA, Cowman AF, et al (2000) clag9: A cytoadherence gene in *Plasmodium falciparum* essential for binding of parasitized erythrocytes to CD36. Proc Natl Acad Sci U S A 97: 4029–4033. 10737759 10.1073/pnas.040561197 PMC18136

[34] Bourke PF, Holt DC, Sutherland CJ, Kemp DJ (1996) Disruption of a novel open reading frame of *Plasmodium falciparum* chromosome 9 by subtelomeric and internal deletions can lead to loss or maintenance of cytoadherence. Mol Biochem Parasitol 82: 25–36. 8943148 10.1016/0166-6851(96)02715-6

[35] Horton P, Nakai K (1997) Better prediction of protein cellular localization sites with the k nearest neighbors classifier. Proc Int Conf Intell Syst Mol Biol 5: 147–152. 9322029

[36] Dingwall C, Laskey RA (1998) Nuclear import: a tale of two sites. Curr Biol 8: R922–924. 9889096 10.1016/s0960-9822(98)00010-4

[37] Thompson JD, Higgins DG, Gibson TJ (1994) CLUSTAL W: improving the sensitivity of progressive multiple sequence alignment through sequence weighting, position-specific gap penalties and weight matrix choice. Nucleic Acids Res 22: 4673–4680. 7984417 10.1093/nar/22.22.4673 PMC308517

[38] Eksi S, Suri A, Williamson KC (2008) Sex- and stage-specific reporter gene expression in *Plasmodium falciparum* . Mol Biochem Parasitol 160: 148–151. 18490066 10.1016/j.molbiopara.2008.04.005 PMC2556552

[39] Patterson S, Robert C, Whittle C, Chakrabarti R, Doerig C, et al (2006) Pre-replication complex organization in the atypical DNA replication cycle of *Plasmodium falciparum*: characterization of the mini-chromosome maintenance (MCM) complex formation. Mol Biochem Parasitol 145: 50–59. 16257456 10.1016/j.molbiopara.2005.09.006

[40] Lopez-Rubio JJ, Mancio-Silva L, Scherf A (2009) Genome-wide analysis of heterochromatin associates clonally variant gene regulation with perinuclear repressive centers in malaria parasites. Cell Host Microbe 5: 179–190. 19218088 10.1016/j.chom.2008.12.012

[41] Vermeulen AN, van Deursen J, Brakenhoff RH, Lensen TH, Ponnudurai T, et al (1986) Characterization of *Plasmodium falciparum* sexual stage antigens and their biosynthesis in synchronised gametocyte cultures. Mol Biochem Parasitol 20: 155–163. 3528848 10.1016/0166-6851(86)90027-7

[42] Gupta SK, Schulman S, Vanderberg JP (1985) Stage-dependent toxicity of N-acetyl-glucosamine to *Plasmodium falciparum* . J Protozool 32: 91–95. 3886901 10.1111/j.1550-7408.1985.tb03020.x

[43] Schmittgen TD, Livak KJ (2008) Analyzing real-time PCR data by the comparative C(T) method. Nat Protoc 3: 1101–1108. 18546601 10.1038/nprot.2008.73

[44] Salanti A, Staalsoe T, Lavstsen T, Jensen AT, Sowa MP, et al (2003) Selective upregulation of a single distinctly structured var gene in chondroitin sulphate A-adhering *Plasmodium falciparum* involved in pregnancy-associated malaria. Mol Microbiol 49: 179–191. 12823820 10.1046/j.1365-2958.2003.03570.x

[45] Schneider P, Schoone G, Schallig H, Verhage D, Telgt D, et al (2004) Quantification of *Plasmodium falciparum* gametocytes in differential stages of development by quantitative nucleic acid sequence-based amplification. Mol Biochem Parasitol 137: 35–41. 15279949 10.1016/j.molbiopara.2004.03.018

[46] Sopko R, Huang D, Preston N, Chua G, Papp B, et al (2006) Mapping pathways and phenotypes by systematic gene overexpression. Mol Cell 21: 319–330. 16455487 10.1016/j.molcel.2005.12.011

[47] Carter R, Graves PM (1988) Gametocytes. In: Wernsdorfer WH, McGregor I, editors. Malaria: Principles and Practice of Malariology. pp. 253–320.

[48] Martinsen ES, Perkins SL, Schall JJ (2008) A three-genome phylogeny of malaria parasites (*Plasmodium* and closely related genera): evolution of life-history traits and host switches. Mol Phylogenet Evol 47: 261–273. 18248741 10.1016/j.ympev.2007.11.012

[49] Pick C, Ebersberger I, Spielmann T, Bruchhaus I, Burmester T (2011) Phylogenomic analyses of malaria parasites and evolution of their exported proteins. BMC Evol Biol 11: 167. 21676252 10.1186/1471-2148-11-167 PMC3146879

[50] Sargeant TJ, Marti M, Caler E, Carlton JM, Simpson K, et al (2006) Lineage-specific expansion of proteins exported to erythrocytes in malaria parasites. Genome Biol 7: R12. 16507167 10.1186/gb-2006-7-2-r12 PMC1431722

[51] Eksi S, Williamson KC (2011) Protein targeting to the parasitophorous vacuole membrane of *Plasmodium falciparum* . Eukaryot Cell 10: 744–752. 21498641 10.1128/EC.00008-11 PMC3127666

[52] Marti M, Good RT, Rug M, Knuepfer E, Cowman AF (2004) Targeting malaria virulence and remodeling proteins to the host erythrocyte. Science 306: 1930–1933. 15591202 10.1126/science.1102452

[53] Hiller NL, Bhattacharjee S, van Ooij C, Liolios K, Harrison T, et al (2004) A host-targeting signal in virulence proteins reveals a secretome in malarial infection. Science 306: 1934–1937. 15591203 10.1126/science.1102737

[54] Bousema T, Okell L, Shekalaghe S, Griffin JT, Omar S, et al (2010) Revisiting the circulation time of *Plasmodium falciparum* gametocytes: molecular detection methods to estimate the duration of gametocyte carriage and the effect of gametocytocidal drugs. Malar J 9: 136. 20497536 10.1186/1475-2875-9-136 PMC2881938

[55] Plotnikov A, Zehorai E, Procaccia S, Seger R (2011) The MAPK cascades: signaling components, nuclear roles and mechanisms of nuclear translocation. Biochim Biophys Acta 1813: 1619–1633. 21167873 10.1016/j.bbamcr.2010.12.012

[56] Major AT, Whiley PA, Loveland KL (2011) Expression of nucleocytoplasmic transport machinery: clues to regulation of spermatogenic development. Biochim Biophys Acta 1813: 1668–1688. 21420444 10.1016/j.bbamcr.2011.03.008

[57] Peter M, Gartner A, Horecka J, Ammerer G, Herskowitz I (1993) FAR1 links the signal transduction pathway to the cell cycle machinery in yeast. Cell 73: 747–760. 8500168 10.1016/0092-8674(93)90254-n

[58] McCord R, Pierce M, Xie J, Wonkatal S, Mickel C, et al (2003) Rfm1, a novel tethering factor required to recruit the Hst1 histone deacetylase for repression of middle sporulation genes. Mol Cell Biol 23: 2009–2016. 12612074 10.1128/MCB.23.6.2009-2016.2003 PMC149475

[59] Jones SK Jr, Bennett RJ (2011) Fungal mating pheromones: choreographing the dating game. Fungal Genet Biol 48: 668–676. 21496492 10.1016/j.fgb.2011.04.001 PMC3100450

[60] Williams P, Winzer K, Chan WC, Camara M (2007) Look who's talking: communication and quorum sensing in the bacterial world. Philos Trans R Soc Lond B Biol Sci 362: 1119–1134. 17360280 10.1098/rstb.2007.2039 PMC2435577

[61] Dyer M, Day KP (2000) Commitment to gametocytogenesis in *Plasmodium falciparum* . Parasitol Today 16: 102–107. 10689328 10.1016/s0169-4758(99)01608-7

[62] Vassella E, Reuner B, Yutzy B, Boshart M (1997) Differentiation of African trypanosomes is controlled by a density sensing mechanism which signals cell cycle arrest via the cAMP pathway. J Cell Sci 110 (Pt 21) 2661–2671. 9427384 10.1242/jcs.110.21.2661

[63] Jeffery GM, Eyles DE (1955) Infectivity to mosquitoes of *Plasmodium falciparum* as related to gametocyte density and duration of infection. Am J Trop Med Hyg 4: 781–789. 13259002 10.4269/ajtmh.1955.4.781

[64] McKenzie FE, Jeffery GM, Collins WE (2007) Gametocytemia and fever in human malaria infections. J Parasitol 93: 627–633. 17626355 10.1645/GE-1052R.1 PMC2483402

[65] Nacher M, Silachamroon U, Singhasivanon P, Wilairatana P, Phumratanaprapin W, et al (2004) Risk factors for *Plasmodium* vivax gametocyte carriage in Thailand. Am J Trop Med Hyg 71: 693–695. 15642956

[66] White-Cooper H, Davidson I (2011) Unique aspects of transcription regulation in male germ cells. Cold Spring Harb Perspect Biol 3: a002626. 21555408 10.1101/cshperspect.a002626 PMC3119912

[67] Metcalf D (2007) Concise review: hematopoietic stem cells and tissue stem cells: current concepts and unanswered questions. Stem Cells 25: 2390–2395. 17690176 10.1634/stemcells.2007-0544

[68] Smalley ME, Brown J (1981) *Plasmodium falciparum* gametocytogenesis stimulated by lymphocytes and serum from infected Gambian children. Trans R Soc Trop Med Hyg 75: 316–317. 7029805 10.1016/0035-9203(81)90348-5

[69] Carter R, Miller LH (1979) Evidence for environmental modulation of gametocytogenesis in *Plasmodium falciparum* in continuous culture. Bull W H O 57 Suppl 1: 37–52. 397008 PMC2395706

[70] Drakeley CJ, Secka I, Correa S, Greenwood BM, Targett GA (1999) Host haematological factors influencing the transmission of *Plasmodium falciparum* gametocytes to Anopheles gambiae s.s. mosquitoes. Trop Med Int Health 4: 131–138. 10206267 10.1046/j.1365-3156.1999.00361.x

[71] Ouedraogo AL, Bousema T, de Vlas SJ, Cuzin-Ouattara N, Verhave JP, et al (2010) The plasticity of *Plasmodium falciparum* gametocytaemia in relation to age in Burkina Faso. Malar J 9: 281. 20939916 10.1186/1475-2875-9-281 PMC3020678

[72] Barnes KI, Little F, Mabuza A, Mngomezulu N, Govere J, et al (2008) Increased gametocytemia after treatment: an early parasitological indicator of emerging sulfadoxine-pyrimethamine resistance in *falciparum* malaria. J Infect Dis 197: 1605–1613. 18471066 10.1086/587645

[73] Price R, Nosten F, Simpson JA, Luxemburger C, Phaipun L, et al (1999) Risk factors for gametocyte carriage in uncomplicated *falciparum* malaria. Am J Trop Med Hyg 60: 1019–1023. 10403336 10.4269/ajtmh.1999.60.1019

[74] Ifediba T, Vanderberg JP (1981) Complete *in vitro* maturation of *Plasmodium falciparum* gametocytes. Nature 294: 364–366. 7031476 10.1038/294364a0

[75] Bossers K, Ylstra B, Brakenhoff RH, Smeets SJ, Verhaagen J, et al (2010) Intensity-based analysis of dual-color gene expression data as an alternative to ratio-based analysis to enhance reproducibility. BMC Genomics 11: 112. 20163706 10.1186/1471-2164-11-112 PMC2838842

[76] Storey JD, Tibshirani R (2003) Statistical significance for genomewide studies. Proc Natl Acad Sci U S A 100: 9440–9445. 12883005 10.1073/pnas.1530509100 PMC170937

[77] Eksi S, Czesny B, Greenbaum DC, Bogyo M, Williamson KC (2004) Targeted disruption of *Plasmodium falciparum* cysteine protease, falcipain 1, reduces oocyst production, not erythrocytic stage growth. Mol Microbiol 53: 243–250. 15225318 10.1111/j.1365-2958.2004.04108.x

[78] Ben Mamoun C, Gluzman IY, Goyard S, Beverley SM, Goldberg DE (1999) A set of independent selectable markers for transfection of the human malaria parasite *Plasmodium falciparum* . Proc Natl Acad Sci U S A 96: 8716–8720. 10411941 10.1073/pnas.96.15.8716 PMC17582

[79] Wu Y, Kirkman LA, Wellems TE (1996) Transformation of *Plasmodium falciparum* malaria parasites by homologous integration of plasmids that confer resistance to pyrimethamine. Proc Natl Acad Sci U S A 93: 1130–1134. 8577727 10.1073/pnas.93.3.1130 PMC40043

[80] Kadekoppala M, Cheresh P, Catron D, Ji DD, Deitsch K, et al (2001) Rapid recombination among transfected plasmids, chimeric episome formation and trans gene expression in *Plasmodium falciparum* . Mol Biochem Parasitol 112: 211–218. 11223128 10.1016/s0166-6851(00)00368-6

[81] Freitas-Junior LH, Hernandez-Rivas R, Ralph SA, Montiel-Condado D, Ruvalcaba-Salazar OK, et al (2005) Telomeric heterochromatin propagation and histone acetylation control mutually exclusive expression of antigenic variation genes in malaria parasites. Cell 121: 25–36. 15820676 10.1016/j.cell.2005.01.037

[82] Silvestrini F, Bozdech Z, Lanfrancotti A, Di Giulio E, Bultrini E, et al (2005) Genome-wide identification of genes upregulated at the onset of gametocytogenesis in *Plasmodium falciparum* . Mol Biochem Parasitol 143: 100–110. 16026866 10.1016/j.molbiopara.2005.04.015

[83] Kooij TW, Carlton JM, Bidwell SL, Hall N, Ramesar J, et al (2005) A *Plasmodium* whole-genome synteny map: indels and synteny breakpoints as foci for species-specific genes. PLoS Pathog 1: e44. 16389297 10.1371/journal.ppat.0010044 PMC1317653

