# Supplementary material for: Plasmodium falciparum Gametocyte Development 1 (Pfgdv1) and Gametocytogenesis Early Gene Identification and Commitment to Sexual Development
Source: PLoS Pathog. 2012 Oct 18;8(10):e1002964. doi: 10.1371/journal.ppat.1002964 (PMC3475683; doi:10.1371/journal.ppat.1002964)
Supplement: Figure S1 — Alignment of PfGDV1 with homologs in other Plasmodium species. The predicted amino acid sequence of Pfgdv1 (PFI1710w) was aligned with homologs identified by BLAST searches of translated sequences on www.PlasmoDB.org, www.ncbi.nlm.nih.gov, and www.sanger.ac.uk/pathogens/malaria using NPS@: Network Protein Sequence Analysis at www.expasy.org. Shown is the CLUSTALW alignment of PfGDV1 (PFI1710w) with Plasmodium reichenowi (Pr332h11.p1k), Plasmodium vivax (PVX_086950), Plasmodium knowlesi (PK_073340), and Plasmodium gallinaceum (Pgal0953d04.p1k) (37). The PfGDV1 sequence and exact matches with other species are highlighted in yellow. Identical amino acids (aa) are shown in red, highly similar aa are green and weakly similar aa are blue. The two helix-rich regions predicted by the GOR4 algorithm are underlined. (DOC) [file ppat.1002964.s001.doc]

**Figure S1. Alignment of PfGDV1 with homologues in other *Plasmodium* species**

**PfGDV1(PFI1710w) MNYKKETCVKLKVKGNSRRRNSAS---R-KMFESWYLYLDNHTKGLFYLNTNDSDTNDAG 56**

**Pr332h11.p1k MNCKKDTCVKLKVKGNSR-----------KMFESWYLYLDNHKKGLFYLNMNDSDTNDGG 49**

**PVX_086950 MENREGRKPDQRVQPRDRRKKSAVQVKRESSKKPWYLHLE-KSELTFFFDLIEEDLTCGF 59**

**PK_073340 MENREGRKSKQLVHPRDRRKKKVMQVKRENSNKPWYLHLE-KSEITFFFDLIEEDLTSRF 59**

**Pgal0953d04.p1k -----------------------------RFKKPWYLQLD-KNDVTYFFDLSEEEIINGG 30**

**PfGDV1(PFI1710w) CEDLVHIYKMKKNELCRHVISLNSEEYAIKKEE--------------------------- 89**

**Pr332h11.p1k CEDLVHIYKMKKNELCRHVISLSSEEYAIKKEE--------------------------- 82**

**PVX_086950 KRKISLATLGSADGSPGEVHDNAFKRVAVKREQRGGVAEGAELDAEVGAEVGAEVGAEVG 119**

**PK_073340 KRKISSTTLGNADGSLDEEHDNAFKRVAVKWEQ---------P----------------- 93**

**Pgal0953d04.p1k KRKMDIDINDNKN-FDKELKENPYKKIAIVKKE--------------------------- 62**

**PfGDV1(PFI1710w) ------------------EAKNNYINI----------EEDVYYN--------SD----IE 109**

**Pr332h11.p1k ------------------EAKNNYINI----------EEDVYYN--------SD----IE 102**

**PVX_086950 AEVVAEVGAEEGAEVCTEVGAEICTEVGAEVCTEVCTESEEKNQEGEDGYLAGEGESSEG 179**

**PK_073340 ----------EDDEVCTDVGDNVSVDA------EMCTETEEKNQ-G-----RGE-DNSEG 130**

**Pgal0953d04.p1k ---------------CDRENENEKKKI----------ESNKINQ--------EN--KKER 87**

**PfGDV1(PFI1710w) QYVDDRKGNSVDLLSSPYIYDRNINN---------------SIGK--------------- 139**

**Pr332h11.p1k QYVDDRKGNSVDLLSSPYIYDRNIKN---------------SICK--------------- 132**

**PVX_086950 LFHVRDENGQSGTLSDDHSFEEHLPGGSRRGVIGSGRAQRGSVQRGSVQRGSVQSGSVQS 239**

**PK_073340 FLNVEGENEGRAGLRDDQPFEGELHG---------------DLRR--------------- 160**

**Pgal0953d04.p1k THVKIKEIDNKESLISTYCYYREINN----------------IRN--------------- 116**

**PfGDV1(PFI1710w) ----------------------------------------------CFCFEMKK------ 147**

**Pr332h11.p1k ----------------------------------------------CFCFEMNK------ 140**

**PVX_086950 GSIHSGSIHSAAIQSGSVQSADVQRRSIHSAGAQSGSVQSLPDEIEPSNYVYRKEPSKLA 299**

**PK_073340 ------------EMT--------------AKNVQSRRVQKWSEEIELSNYVYRKGSFEDT 194**

**Pgal0953d04.p1k --------------------------------------------KLVTNYNITK------ 126**

**PfGDV1(PFI1710w) --S------FNSLMYLDNHLSYFYGNKKEEMGNFLQEYNKKRFEDVCNRGLLNNFKSDIY 199**

**Pr332h11.p1k --S------FNSLMYLDNHLSYFYGNKKEEMGKFLQEYNKKRFEDVCNRGLLNNFKSDIY 192**

**PVX_086950 MRCTHYKKPYNLLLYLNAFLRELTCNKKEAVDRLLKAFNRKRFNNKNNKGMRNNFKGDLY 359**

**PK_073340 MRCTQNKMPYNLLLYLNTFLGDLACN-KDTMARWLEEFNKKRFNNRNNKGMRNNFKADLY 253**

**Pgal0953d04.p1k --S------YNLFLYLNMFAKYLNXSKKTSVDKFXRNFNKRRFNNSNNRGLFNNYKTDIY 178**

**PfGDV1(PFI1710w) EIGIYEENIVSFKLLDCPNRHLIKNTYYMNADLIFNEIHNLLNKMYPLKHIMNNHHLVKK 259**

**Pr332h11.p1k EIGIYEENIVSFKLLDCQNRHLIKNTYYMNADLIFNEIHNLLNKMYPLKHIMNNHHLVKK 252**

**PVX_086950 RVVIHAGGILSFKLLKNNN-EIGKKTYYMNEELLHLEINNILGKNYPLKYIFYMNQEVRD 418**

**PK_073340 RVVIHDGGIISFKLITSRN-EIGKTTYYMNEELLHLEINNILEKYYPLKYIFYLNQKVRD 312**

**Pgal0953d04.p1k EIGIYEKNIFTFKLLKKNK-KIGNKIYYVSEELLNLEIDNFLAKFYPIKYIVKDNPLIKN 237**

**PfGDV1(PFI1710w) YQKKVPE-HLKMEKALKFGIVLDFDYVRENFENNLKKTMTLLDFLIILDKIWEIFRQNCV 318**

**Pr332h11.p1k YQKKVPE-HLKMEKALKFGIVLDFDYVRENFENNLKKTMTLLDFLIILDKIWEIFRQNCV 311**

**PVX_086950 FQRVVMPLELRSDKPITCGILLDFDYMGRSFEMNEQRPMKLVDLVLMLDKICEVLRENCT 478**

**PK_073340 FQKLVIPLELKSDKPITCGILLDFDYMGHSFEMIKKRPMKLVDLMVILDKICELLREKCT 372**

**Pgal0953d04.p1k YQRQINP-NLSKEKPLTFGLVLDFDYIRECFEQNEERSMELLDFIVILDKISEVFRENCL 296**

**PfGDV1(PFI1710w) IMFLHVCIFK---------------------------------------DNDTNKDINN- 338**

**Pr332h11.p1k IMFLHVCIFK---------------------------------------DNDTNKDINN- 332**

**PVX_086950 VIYVHHAGVESDREVQGSAVVASTSGTASTSGAVNTSGAVNTSDAANTSGAANTSGAANT 538**

**PK_073340 VIYVHYARR-S---------IP--Q------------------------GEANSAGAAN- 396**

**Pgal0953d04.p1k IIYLHISIF----------------------------------------SENGIFEGIN- 316**

**PfGDV1(PFI1710w) ----------------------------------HRKRYSYMYEDLKNTLDLLKQKGIPV 364**

**Pr332h11.p1k ----------------------------------HRKRYSYIYEDLKNTLHLLKQKGIPV 357**

**PVX_086950 SEAANTSGAAELILPHPRRGAKHTANAHRGHACRHKKAYGYLFDDLESTLKLFEERNIKV 598**

**PK_073340 ----------ELILSHPRRAPKNTANAYRGHACRHERSYSYLFDDLQSTLELFEERNIKM 445**

**Pgal0953d04.p1k --------------------------IYR------N-TXEYLFEGLKSTTKLFEERNINL 342**

**PfGDV1(PFI1710w) VMRINP-----------------------------FQSFR-----LFNHNEFSNPFIQDV 390**

**Pr332h11.p1k VMRINP-----------------------------FQSFR-----LFNHNEFSNPFIQDV 383**

**PVX_086950 VIRTSG-----------------------------------------ARAKKKSKLVKDM 617**

**PK_073340 VIRTSEFTEEVEMTSSAADTMLSSRSFRPSPPTCLPPTSHPSAIADASQKKKKKTIVKSI 505**

**Pgal0953d04.p1k ITRVHP-----------------------------LQSYR----KIIIDSEMDNPMIKDV 369**

**PfGDV1(PFI1710w) NDLYMNE--------------RVDHIILVTNDSDVISYSYHFDHTIIYCIKDKYKNVTNV 436**

**Preichenowi1710w NDLYMNE--------------QVDHIILVTNDSDVISYSYNFDHTIIYCIKDKYKNATSV 429**

**PVX_086950 AQLFQNP--------------LVDNILLLCNDVDVISYCYHVRHRIKSKSG--------- 654**

**PK_073340 AQLFENP--------------YVDNVLLLCNDIDVISYCYHVRHKVKYKNG--------- 542**

**Pgal0953d04.p1k NKLFENK--------------EIDNIFLITNDIDVISYCYKSRHKIKFK----------- 404**

**PfGDV1(PFI1710w) PNINSDINVDTITHPETTNNISNHYKNHNTIMFKKPVILICSLNNLPLKNNKIHDDIILD 496**

**Pr332h11.p1k HNINTDITVDTITHPETTNNISNHYKNHNTIMFKKPVILICSLNNLPLKNNKIHDDIILD 489**

**PVX_086950 ---------------------EACMKEDYTFQFMKPVLIFSFLNNLPIKEGKIYPHLKLD 693**

**PK_073340 ---------------------EVSMKKEYAFRFMKPVLIFCFLNNLPVKNGKIYPHLKLD 581**

**Pgal0953d04.p1k ---------------------EKNKQNDYIISYKKPVIVLSFLNNLPIKNNKIHPYLKLD 440**

**PfGDV1(PFI1710w) DFCYMTYIIKTLYFRFQSRSIRNMLKSNLNNTTNFVINRITQMNNTNRLLKNKINVLLLD 556**

**Pr332h11.p1k DFCYMTYIIRTLYFRFQSRSIRNMLKSNLNNTTNFVINRITQMNNANRLIKSKINVLLLD 549**

**PVX_086950 RFCYMTFIITTYLLRCQARNLQKLATE-SHFRVNLVEDLISKYNHENRNLKKRMNVLLLD 752**

**PK_073340 RFCYMSFIIRTYLLRCQANKLQRLATE-IHFNVDLVQELIKKYNMENSTLKKTMNILLLD 640**

**Pgal0953d04.p1k KFCYITFIIKSLYLRCQAKNIENIKMN-SKENKNLIEKYITMYNIKNKKLKKKINVILLD 502**

**PfGDV1(PFI1710w) DIIYKIRP-----RKEN------KNVSIHHLLQKAYSPFYYPIHYYIKEKCTYK 599**

**Pr332h11.p1k DIIYKIKP-----RKEN------KNVSIHHLLQKTYSPFYYPIHYYIKEKCTYK 592**

**PVX_086950 DLLYKVSAKCKTKKKAKKRAEADAEVSVSDLLRRSFSPVYYPMHYYLEES---- 802**

**PK_073340 DLLYKMKG--KTKKKAH------AEIPLSELMRRSFSAVYYPMQYYLQDS---- 682**

**Pgal0953d04.p1k DIIYKINF-----KNQN------TIIKVSSLLKRTFLPFYYPIHYYIKEN---- 541**
