# Supplementary figures and images for: Plasmodium falciparum Gametocyte Development 1 (Pfgdv1) and Gametocytogenesis Early Gene Identification and Commitment to Sexual Development
Source: PLoS Pathog. 2012 Oct 18;8(10):e1002964. doi: 10.1371/journal.ppat.1002964 (PMC3475683; doi:10.1371/journal.ppat.1002964)

Fig. S2

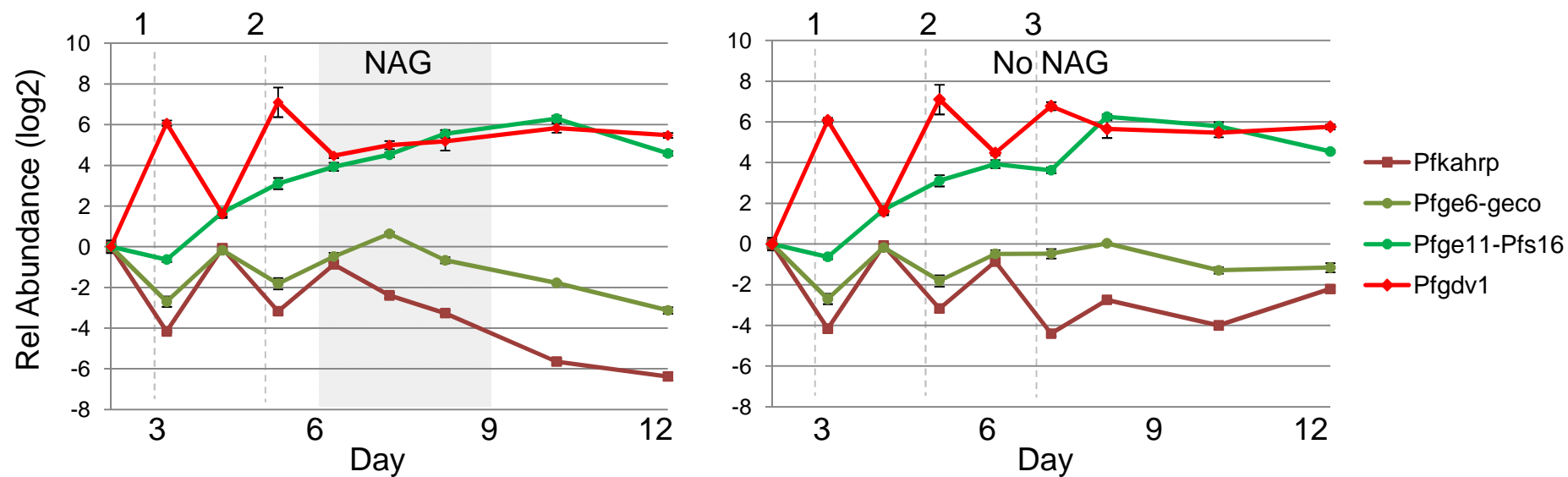

Supplement: Figure S2 — Expression profile of Pfge6 ( Pfgeco ) through gametocytogenesis. MACS purified late stage parasite cultures were set up at 6% hematocrit and sorbitol synchronized 2 hours later to remove all but the newly invaded ring stage parasites. The relative abundance of the RNA corresponding to the indicated gene in relation to the seryl tRNA synthetase ratio on day two is graphed: Pfkahrp (brown), Pfge6-geco (olive green), Pfge11-Pfs16 (green) and Pfgdv1 (bright red). The 3 asexual cycles are indicated by numbers as well as gray dotted lines and NAG treatment is indicated by the gray box. Data from one of three independent experiments is shown. The samples from the different time points were tested in triplicate and the average relative expression is plotted with the error bars representing the range. (PDF) [file ppat.1002964.s002.pdf]
