# Supplementary material for: Plasmodium falciparum Gametocyte Development 1 (Pfgdv1) and Gametocytogenesis Early Gene Identification and Commitment to Sexual Development
Source: PLoS Pathog. 2012 Oct 18;8(10):e1002964. doi: 10.1371/journal.ppat.1002964 (PMC3475683; doi:10.1371/journal.ppat.1002964)
Supplement: Table S1 — Genes differentially expressed in the 3D7.G+ and 3D7.Gdef clones. (DOC) [file ppat.1002964.s003.doc]

**Table S1: Genes differentially expressed in the 3D7.G+ and 3D7.G_def_ clones**

|  |  |  |  | Comparative Microarray  3D7.G+/3D7.G_def_ Signal Ratio | | | | |  |
| --- | --- | --- | --- | --- | --- | --- | --- | --- | --- |
|  |  |  |  | 1.4/0.9 %  Parasitemia | |  | 5.2/5.5 %  Parasitemia | |  |
| *Pfge* | Oligo ID | Common Name | PlasmoDB ID | Avg | SEM |  | Avg | SEM | Product  Description |
| 1 | N129_5 | *Pfge1** | [PF14_0744](http://plasmodb.org/plasmo/showRecord.do?name=GeneRecordClasses.GeneRecordClass&project_id=PlasmoDB&source_id=PF14_0744) | 278.9 | 135.7 |  | 217.7 | 64.4 | Exported |
| 2 | N129_1 | *Pfge2* | [PF14_0745](http://plasmodb.org/plasmo/showRecord.do?name=GeneRecordClasses.GeneRecordClass&project_id=PlasmoDB&source_id=PF14_0745) | 138.2 | 35.0 |  | 152.6 | 44.0 | Probable |
| 3 | N139_24 | *Pfge3** | [PF14_0748](http://plasmodb.org/plasmo/showRecord.do?name=GeneRecordClasses.GeneRecordClass&project_id=PlasmoDB&source_id=PF14_0748) | 122.3 | 16.6 |  | 214.8 | 74.0 | Exported (PHISTa) |
| 4 | I13417_1 | *Pfgdv1*^o^ | [PFI1710w](http://plasmodb.org/plasmo/showRecord.do?name=GeneRecordClasses.GeneRecordClass&project_id=PlasmoDB&source_id=PFI1710w) | 69.2 | 12.8 |  | 39.9 | 11.0 | Cytoadherence linked |
| 5 | M32775_1 | *Pfg27** | [PF13_0011](http://plasmodb.org/plasmo/showRecord.do?name=GeneRecordClasses.GeneRecordClass&project_id=PlasmoDB&source_id=PF13_0011) | 43.1 | 7.8 |  | 59.1 | 15.9 | Gamete antigen 27/25 |
| 6 | oPFL0057 | *Pfgeco** | [PFL2550w](http://plasmodb.org/plasmo/showRecord.do?name=GeneRecordClasses.GeneRecordClass&project_id=PlasmoDB&source_id=PFL2550w) | 37.8 | 2.1 |  | 55.5 | 7.3 | DNAJ |
| 7 | N129_17 | *Pfge7* | [PF14_0736](http://plasmodb.org/plasmo/showRecord.do?name=GeneRecordClasses.GeneRecordClass&project_id=PlasmoDB&source_id=PF14_0736) | 27.9 | 2.0 |  | 34.5 | 8.3 | Exported |
| 8 | N129_20 | *Pfge8* | [PF14_0735](http://plasmodb.org/plasmo/showRecord.do?name=GeneRecordClasses.GeneRecordClass&project_id=PlasmoDB&source_id=PF14_0735) | 18.0 | 2.9 |  |  |  | Probable |
| 9 | M23550_7 | *msp7-5* | [PF13_0196](http://plasmodb.org/plasmo/showRecord.do?name=GeneRecordClasses.GeneRecordClass&project_id=PlasmoDB&source_id=PF13_0196) | 12.3 | 1.6 |  | 11.7 | 1.2 | MSP7-like |
| 10 | M21439_2 | *Pf47** | [PF13_0248](http://plasmodb.org/plasmo/showRecord.do?name=GeneRecordClasses.GeneRecordClass&project_id=PlasmoDB&source_id=PF13_0248) | 11.8 | 1.4 |  | 21.4 | 4.4 | 6-cysteine |
| 11 | D49176_46 | *Pfs16** | [PFD0310w](http://plasmodb.org/plasmo/showRecord.do?name=GeneRecordClasses.GeneRecordClass&project_id=PlasmoDB&source_id=PFD0310w) | 11.2 | 1.4 |  | 14.8 | 0.9 | Sexual stage-specific |
| " | oPFD6695 | " | " | 11.4 | 0.8 |  | 17.3 | 0.9 |  |
|  | N134_107 |  | [PF14_0588](http://plasmodb.org/plasmo/showRecord.do?name=GeneRecordClasses.GeneRecordClass&project_id=PlasmoDB&source_id=PF14_0588) | 9.4 | 0.5 |  | 12.0 | 1.9 | Conserved |
|  | N138_36 |  | [PF14_0290](http://plasmodb.org/plasmo/showRecord.do?name=GeneRecordClasses.GeneRecordClass&project_id=PlasmoDB&source_id=PF14_0290) | 8.5 | 0.6 |  | 11.8 | 1.0 | Conserved |
|  | F61856_1 | surfin | [PF08_0002](http://plasmodb.org/plasmo/showRecord.do?name=GeneRecordClasses.GeneRecordClass&project_id=PlasmoDB&source_id=PF08_0002) | 7.2 | 0.3 |  | 8.2 | 1.3 | SURFIN8.2 |
|  | F20166_1 | " | " | 6.6 | 0.1 |  | 7.5 | 0.4 |  |
|  | N142_5 |  | [PF14_0708](http://plasmodb.org/plasmo/showRecord.do?name=GeneRecordClasses.GeneRecordClass&project_id=PlasmoDB&source_id=PF14_0708) | 6.9 | 0.2 |  | 11.5 | 2.4 | Probable |
|  | C445 |  | [PFC0680w](http://plasmodb.org/plasmo/showRecord.do?name=GeneRecordClasses.GeneRecordClass&project_id=PlasmoDB&source_id=PFC0680w) | 6.5 | 0.9 |  | 13.3 | 1.7 | Conserved |
|  | L2_280 | *Pfmdv1/Pfeg3** | [PFL0795c](http://plasmodb.org/plasmo/showRecord.do?name=GeneRecordClasses.GeneRecordClass&project_id=PlasmoDB&source_id=PFL0795c) | 6.4 | 0.2 |  | 12.5 | 0.5 | Male development 1 |
|  | Ks75_18 |  | [PF11_0038](http://plasmodb.org/plasmo/showRecord.do?name=GeneRecordClasses.GeneRecordClass&project_id=PlasmoDB&source_id=PF11_0038) | 6.2 | 0.5 |  | 10.4 | 0.2 | Exported |
|  | F47255_2 |  | [PF08_0033](http://plasmodb.org/plasmo/showRecord.do?name=GeneRecordClasses.GeneRecordClass&project_id=PlasmoDB&source_id=PF08_0033) | 6.0 | 0.5 |  | 6.7 | 1.3 | Membrane skeletal IMC1 |
|  | N145_40 |  | [PF14_0010](http://plasmodb.org/plasmo/showRecord.do?name=GeneRecordClasses.GeneRecordClass&project_id=PlasmoDB&source_id=PF14_0010) | 0.2 | 0.0 |  | 0.2 | 0.0 | Glycophorin binding |

Comparative genomic microarray analysis of RNA isolated from sorbitol-synchronized 3D7.G+ and 3D7.G_def_ clones at 1.4% and 0.9% parasitemia, respectively, and then again 2 days later at 5.2% and 5.5% parasitemia, respectively. These parasitemias were reached 4 and 6 days after sorbitol-synchronized asexual parasites were used to set up gametocyte cultures at 0.1% parasitemia.

Oligonucleotides (Oligo ID) corresponding to the *Pfge* genes and those with a mean *Pfgvd1* ± ratio > 5 or < 0.2 at both time point are listed in descending order, along with their corresponding common name or multigene family, PlasmoDB annotated gene designation (PlasmoDB ID), and current PlasmoDB product description. In the Common Name column, *Pfgdv1—*which is not present in the gametocyte deficient line—is indicated with ^o^, and known gametocyte-associated genes are indicated by * . The PlasmoDB ID is hyperlinked to the corresponding gene at [www.plasmoDB.org](http://www.plasmoDB.org/).

**References**

1. Alano P, Premawansa S, Bruce MC, Carter R (1991) A stage specific gene expressed at the onset of gametocytogenesis in Plasmodium falciparum. Mol Biochem Parasitol 46: 81-88.

2. Bruce MC, Carter RN, Nakamura K, Aikawa M, Carter R (1994) Cellular location and temporal expression of the Plasmodium falciparum sexual stage antigen Pfs16. Mol Biochem Parasitol 65: 11-22.

3. Young JA, Fivelman QL, Blair PL, de la Vega P, Le Roch KG, et al. (2005) The Plasmodium falciparum sexual development transcriptome: a microarray analysis using ontology-based pattern identification. Mol Biochem Parasitol 143: 67-79.

4. Silvestrini F, Bozdech Z, Lanfrancotti A, Di Giulio E, Bultrini E, et al. (2005) Genome-wide identification of genes upregulated at the onset of gametocytogenesis in Plasmodium falciparum. Mol Biochem Parasitol 143: 100-110.

5. van Schaijk BC, van Dijk MR, van de Vegte-Bolmer M, van Gemert GJ, van Dooren MW, et al. (2006) Pfs47, paralog of the male fertility factor Pfs48/45, is a female specific surface protein in Plasmodium falciparum. Mol Biochem Parasitol 149: 216-222.

6. Furuya T, Mu J, Hayton K, Liu A, Duan J, et al. (2005) Disruption of a Plasmodium falciparum gene linked to male sexual development causes early arrest in gametocytogenesis. Proc Natl Acad Sci USA 102: 16813-16818.
