# Supplementary material for: Plasmodium falciparum Gametocyte Development 1 (Pfgdv1) and Gametocytogenesis Early Gene Identification and Commitment to Sexual Development
Source: PLoS Pathog. 2012 Oct 18;8(10):e1002964. doi: 10.1371/journal.ppat.1002964 (PMC3475683; doi:10.1371/journal.ppat.1002964)
Supplement: Table S2 — Clinical and hematological characteristics of the patient cohort. (DOCX) [file ppat.1002964.s004.docx]

**Table S2: Clinical and hematological characteristics of the patient cohort.**

n 20

Male/female 16/4

Average age, yr 26.6 (15-40)

0/A/B/AB 8/5/4/3

Systolic BP, mmHg 103 (69-128)

Diastolic BP mmHg 63.2 (47-77)

Resp Rate per min 22.8 (16-28)

Heart Rate per min 97 (57-121)

Oral Temp, ^o^C 38 (36.2-40)

Hematocrit, % 36.8 (25-48)

Parasitemia, % 0.18 (0.01-0.37)

Gametocytemia, % 0.011 (0.002-0.029)

The number of male and female patients and the number of patients with A/B/AB/O blood type in the total population are indicated, as is the average (range) measurements for the other clinical parameters.
