# Supplementary material for: Plasmodium falciparum Gametocyte Development 1 (Pfgdv1) and Gametocytogenesis Early Gene Identification and Commitment to Sexual Development
Source: PLoS Pathog. 2012 Oct 18;8(10):e1002964. doi: 10.1371/journal.ppat.1002964 (PMC3475683; doi:10.1371/journal.ppat.1002964)
Supplement: Table S3 — Oligonucleotide primers. (DOCX) [file ppat.1002964.s005.docx]

**Table S3: Oligonucleotide primers**

**Chromosome 9 deletion mapping**: The oligonucleotide primers listed were used for the 8 PCRs depicted

in Fig. 2A & B. Their position on chromosome 9 is included in parentheses.

Primer set 1

Chr9.1.forward (chr9:1,373,822): 5’-GCA GAA CGT GTT TTT ACA CAA ATA AAA TA-3’

Chr9.1.reverse (chr9:1,374,469): 5’-AAT ATT GAA CTA TGC CAT CAT ATC ATA GG - 3’

Primer set 2

Chr9.2.forward (chr9:1,375,899): 5’-TGC GTA AAT TAT TTT ATT TTT GAA ACC-3’

Chr9.2.reverse (chr9:1,377,781): 5’-GAT GAG TTT AAA TTG GAA GGG CTA T-3’

Primer set 3

Chr9.3.forward (chr9:1,380,204): 5’-AAG CAC AAA TAA AAA CAT TTT CCA A-3’

Chr9.3.reverse (chr9:1,381,981): 5’-TTT GTT TTG TGT TCA TAA TTC TAA AGT TC-3’

Primer set 4

Chr9.4.forward (chr9:1,384,030): 5’-CAT GAG GGA AAT TTA ATC AAA ATC A-3’

Chr9.4.reverse (chr9:1,385,907): 5’-AAA TAA AGT AAT TGA TGG TCT TTT TGG-3’

Primer set 5

Chr9.5.forward (chr9:1,388,129): 5’-TGT ATA TAT GTT TTT ACA TGT GAT CGT TT-3’

Chr9.5.reverse (chr9:1,390,043): 5’-TCA TAT TTT TAT TTG ATG AAC ATT CTT ATG-3’

Primer set 6

Chr9.6.forward (chr9:1,392,099): 5’-TGA TTC GAA TTA ATG CTT TAA AAA TG-3’

Chr9.6.reverse (chr9:1,392,640): 5’-ATG TAT AAA TAC ATG AAC GTG TTC AC-3’

Primer set 7

Chr9.7.forward (chr9:1,396,120): 5’-CCA TAT TCG ATT ATT ATT TTG CCT TT-3’

Chr9.7.reverse (chr9:1,397,997): 5’-AAA AAT GAT AAC CTC ACA AAT GTA CG-3’

Primer set 8

Chr9.8.forward (chr9:1,400,400): 5’-CAA TAG CAA CCT TTC TTA TAT GCT TTC-3’

Chr9.8.reverse (chr9:1,401,583): 5’-TGG TTC TAA CCT TTT CGT TCT TCG TG-3’

***Pfgdv*1 complementation and reporter-tagging constructs:** The oligonucleotide primers listed were used

to generate complementation and GFP and HA reporter constructs.

**Complementation:**

*Pfgdv1*.5’FR.-1236.forward: 5’-TAAT CCGCGG GAA TAA AAT GTG ACA TAT TTT CGT-3’

*Pfgdv1*.5’FR.1.reverse: 5’-TAAT TCTAGA ATT AAT AAT ATG GAA GTT ATA TAA ATA-3’

*Pfgdv1*.1.forward.*Xba*I: 5’-TAAT TCTAGA AGT AAC TAC AAA AAA GAG ACT TGC-3’

*Pfgdv1*.1800.reverse.*Xba*I: 5’-TAAT TCTAGA TTA TTT ATA TGT ACA TTT TTC TTT TAT-3’

***Pfgdv1* reporter tagging**:

*Pfgdv1*.1.forward.*Spe*I: 5’-TAAT ACTAGT AGT AAC TAC AAA AAA GAG ACT TGC-3’

*Pfgdv1*.1800.reverse.*Bam*HI: 5’-TAAT GGATCC TTA TTT ATA TGT ACA T TT TTC TTT TAT-3’

HA.forward 5’-CTAGA ATG TAC CCA TAC GAC GTC CCA GAC TAC GCT TAC CCA TAC GAC GTC CCA GAC TAC GCT TAC

CCA TAC GAC GTC CCA GAC TAC GCT ACTAGT GCA G-3’

HA.reverse 5’-gatcc TGC ACTAGT agc gta gtc tgg gac gtc gta tgg gta AGC gta gtc tgg gac gtc gta tgg

gta AGC gta gtc tgg gac gtc gta tgg gta CATT-3’

*Pfgdv1*.1.forward.*Xba*I: 5’-TAAT TCTAGA AGT AAC TAC AAA AAA GAG ACT TGC-3’

*Pfgdv1*.1797.reverse.*Spe*I: 5’-TAAT ACTAGT TTT ATA TGT ACA TTT TTC TTT TAT-3’

GFP.*Spe*I.forward 5’-TAAT ACTAGT CAT AGA TCT AAA GGA GAA GAA CTT-3’

GFP.stop.*Bam*HI.reverse 5’-TAAT GGATCC TTA TTT GTA AGT TTC ATC CAT GCC-3’

***Pfgdv1* confirmation:** The oligonucleotide primers listed were used to confirm the presence of *Pfgdv1* in gDNA

as shown in Fig. 2A.

*Pfgdv1*.forward 5’- TTG GAT TGT CCT AAT CGT CAT C-3’

*Pfgdv1*.reverse 5’-TCC TTC CTG GGC CTA ATT TT-3’

seryl-tRNA synthetase.forward 5’-AAG TAG CAG GTC ATC GTG GTT-3’

seryl-tRNA synthetase.reverse 5’-TTC GGC ACA TTC TTC CAT AA-3’

***qRT-PCR***:

*Seryl tRNA sythetase* (PF07_0073) forward 5’-AAGTAGCAGGTCATCGTGGTT-3’

*Seryl tRNA sythetase* (PF07_0073) reverse 5’-GTTCGGCACATTCTTCCATAA-3’

*Adolase* (PF14_0425) forward 5’-CAGCAGATGTTGCCGAAGAAT

*Adolase* (PF14_0425) reverse 5’-TTCCCTTTCCAGCTTGAACAA-3’

*Knob associated histidine rich protein* (PFB0100c) forward 5’-CATGGTGCAGGCTATTCAG-3’

*Knob associated histidine rich protein* (PFB0100c) reverse 5’-TTCACCGTCATTTCCTTCATGC-3’

*Pfgdv1 (*PFI1710w) forward 5’-TAGGCGTCGAAATAGTGCTAGTAGAAA-3’

*Pfgdv1 (*PFI1710w) reverse 5’-GTCCTCACAACCAGCATCATTAGTA-3’

*Pfg14.744* (*Pfge1,* PF14_0744) forward 5’-CTTATGGCTACAAGCATTTGCT-3’

*Pfg14.744* (*Pfge1,* PF14_0744) reverse 5’-CTTGAGCCAATTCAACATTACAG-3’

*Pfg14.745* (*Pfge2,* PF14_0745) forward 5’-TGTGGTGCATTATGGGA-3’

*Pfg14.745* (*Pfge2,* PF14_0745) reverse 5’-GTTCAGTATAAAAATATCGAGCATC-3’

*Pfg14.748* (*Pfge3,* PF14_0748) forward 5’-AAGGGTAGTTCCTAGAGCAGT-3’

*Pfg14.748* (*Pfge3,* PF14_0748) reverse 5’-CAGCTTTATCAGAATTCCCATTGT-3’

*Pfg27* *(Pfge5,* PF13_0011) forward 5’-AAGCCCTTGGATAAATTTGGAA-3’

*Pfg27* *(Pfge5,* PF13_0011) reverse 5’-CATGAAACTCAATATCCCCAACTTT-3’

*Pfgeco* (*Pfge6,* PFL2550w) forward 5’-GAAAACGTGTGTTATCGTTCAA-3’

*Pfgeco* (*Pfge6,* PFL2550w) reverse 5’-CCATTAAAAGTACTCATTCCGCTA-3’

*Pfg14.736* (*Pfge7,* PF14_0736) forward 5’-ACAAAATCAAGAATCAAGTAATACAAGT-3’

*Pfg14.736* (*Pfge7,* PF14_0736) reverse 5’-CTTCTATAGTAATATTGAGGAATCGTGTA-3’

*Pfg14.735* (*Pfge8,* PF14_0735) forward 5’-GTTGATTCACCACTTCGATTC-3’

*Pfg14.735* (*Pfge8,* PF14_0735) reverse 5’-TCTATCTCATATTTACCTATAGCATCG-3’

*Merozoite surface protein 7-like* (*Pfge9,* PF13_0196) forward 5’-TACCAGGTGCCTTATCAAGTG-3’

*Merozoite surface protein 7-like* (*Pfge9,* PF13_0196) reverse 5’-CTTGGTTGTGATTCGGTTGATG-3’

*Pfs47* (*Pfge10,* PF13_0248) forward 5’-GTGTATGGGAAGAATGATCAGC-3’

*Pfs47* (*Pfge10,* PF13_0248) reverse 5’-GTGTTGAGCTTAATAGTTCACTAATAC-3’

*Pfs16 (Pfge11,* PFD0310w) forward 5’-CAAAAAAGCCCGCTGGAA-3’

*Pfs16* *(Pfge11,* PFD0310w) reverse 5’-CCTTGAGATAGTCCACCTTGATTAGG-3’
